# Glycerol-Vanillin Acetals as Green and Efficient Antioxidants

**DOI:** 10.1021/acsomega.6c03930

**Published:** 2026-06-12

**Authors:** Daniella R. Fernandes, Júlio C. G. de Almeida, Nilton Rosenbach, Claudio J. A. Mota

**Affiliations:** † 341411 Universidade Federal do Rio de Janeiro, Instituto de Química. Av. Athos da Silveira Ramos 149, CT Bl A, 21941-909 Rio de Janeiro, Brazil; ‡ Faculdade de Ciência Exatas e Engenharias, 28130 Universidade do Estado do Rio de Janeiro, Av. Manuel Caldeira de Alvarenga 1203, Rio de Janeiro 23070-200, Brazil; § Universidade Federal do Rio de Janeiro, Escola de Química, Av. Athos da Silveira Ramos 149, CT Bl E, Rio de Janeiro 21941-909, Brazil

## Abstract

Acetals were synthesized upon the acid-catalyzed reaction of glycerol with vanillin, ethyl vanillin, cinnamaldehyde, and 1-napthaldehyde using Amberlyst-15 as a solid acid catalyst. The acetals were successfully isolated, characterized, and evaluated for their antioxidant activity using the DPPH radical scavenging assay. The antioxidant efficiency, measured as the effective concentration (EC_50_), was 2.1 mol per mol of DPPH for the ethyl vanillin-glycerol acetal (EVGA) and 5.5 mol per mol of DPPH for VGA, significantly superior to the values found for the other glycerol acetals. These values are close to BHT, a fossil-derived commercial antioxidant, which showed an EC_50_ of 1.0 mol per mol of DPPH under the same test conditions. Vanillin and ethyl vanillin presented EC_50_ values of 21.5 and 32.6, respectively. EVGA and VGA were tested as antioxidants in soybean biodiesel, presenting an increased induction period in the racimat test relative to the neat biodiesel. DFT calculations showed a decrease in the bond dissociation energy (BDE) of the phenolic OH of EVGA and VGA with respect to vanillin and ethyl vanillin, explaining their superior antioxidant performance. In addition, the methine C–H bond of the acetal group could potentially act as an antioxidant center according to DFT calculations. EVGA and VGA acetals are promising green antioxidants, obtained from biomass-derived raw materials, that could be used in food, fuel, and materials applications, as substitutes for BHT.

## Graphical abstract

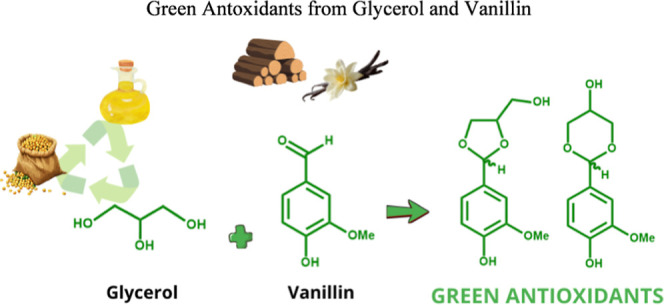

## Introduction

The use of renewable raw materials has been a key strategy for promoting sustainable and circular chemistry. In this context, glycerol, a byproduct of biodiesel production, appears as one of the most promising renewable platform chemicals.^1–3^ Biodiesel is usually produced from the transesterification of vegetable oils.^4^ This process yields approximately 10 wt % of glycerol, and the economic feasibility of biodiesel production requires the valorization of glycerol.

Numerous catalytic transformations of glycerol have been explored as strategies to valorize this renewable feedstock and contribute to a transition toward a more sustainable energy matrix. They encompass oxidation,^5,6^ reforming,^7,8^ hydrogenolysis,^9,10^ etherification,^11,12^ esterification,^13,14^ and acetalization/ketalization^15,16^ of glycerol, among others.

We extensively studied the conversion of glycerol into value-added products. Glycerol acetylation with acetic acid and acetic anhydride affords the acetins.^17,18^ Etherification of glycerol with ethanol yields ethers that can be used as additives to improve the flowing properties of biodiesel.^19^ Solketal, produced from the acid-catalyzed reaction of glycerol with acetone,^12,20^ has been shown to improve the octane number and reduce gum formation in blends with gasoline,^22^ whereas glycerol carbonate could be obtained from the direct reaction of CO_2_ with glycerol in the presence of metal oxide catalysts.^23,24^ A selective process of glycerol hydrogenation to propene has also been developed, upon the use of Fe–Mo catalysts impregnated on activated carbon.^25^ The reaction can be run in continuous flow with 100% conversion and up to 90% selectivity to propene.

The incorporation of antioxidant additives is essential to mitigate oxidative degradation and ensure the long-term storage stability of the main product. They find applications in many sectors, such as food,^26^ fuels,^27,28^ and plastics,^29^ among others. Conventional commercial antioxidants, such as butylated hydroxyanisole, butylated hydroxytoluene (BHT), *tert*-butylhydroquinone, trihydroxybutylphenone, and alkyl gallates (propyl, octyl, and dodecyl gallates), are predominantly derived from nonrenewable resources. For instance, BHT is normally produced from the acid-catalyzed alkylation of *p*-cresol with isobutylene^30^ and cannot be considered to be green or sustainable. Moreover, most of these antioxidants have been associated with toxicological problems.^31,32^

Vanillin (4-hydroxy-3-methoxybenzaldehyde) is an important natural product used as a fragrance in food, perfume, and pharmaceutical sectors. It is the primary component of the vanilla extract. Today, the demand for vanillin exceeds the production from natural extraction, and there are many methods of its industrial synthesis, including biotechnological routes.^33^ Ethyl vanillin (3-ethoxy-4-hydroxybenzaldehyde) is mostly obtained by synthetic routes from guaethol or sassafras oil.^34^ Vanillin and ethyl vanillin can also be prepared from lignin^35^ or other biomass sources,^36^ being important renewable raw materials for producing chemicals.

We have studied the acetalization of glycerol with benzaldehyde, anisaldehyde, and furfural using an Amberlyst-15 acid catalyst.^37^ The produced acetals exhibited radical-scavenging activity in the diphenyl-picryl-hydrazyl (DPPH) assays, especially the anisaldehyde–glycerol acetal, named (AGA). However, none of them showed great potential to replace traditional antioxidants, such as BHT, as the EC_50_ was significantly higher. AGA presented the best antioxidant performance among the three glycerol acetals studied. This fact was attributed to the formation of a delocalized free radical upon the abstraction of the methine hydrogen atom. Thus, the *p*-OCH_3_ group of the anisaldehyde would provide additional resonance stabilization when compared with that of benzaldehyde. None of the former glycerol acetals studied presented phenolic OH groups, which are normally present in commercial antioxidants, such as BHT.

Therefore, this study is aimed at exploiting the acetalization of glycerol with other aromatic aldehydes to either include phenolic OH groups or provide an enhanced resonance delocalization of the free radical formed upon the abstraction of the methine hydrogen atom. To cope with the first objective, we synthesized acetals of vanillin-glycerol (VGA) and ethyl vanillin-glycerol (EVGA), both presenting phenolic OH (Figure 1). Indeed, vanillin and ethyl vanillin present antioxidant activity,^38–40^ being valuable food additives.^41^ To cope with the second objective of increasing the antioxidant activity by increasing the delocalization of the free radical, we synthesized acetals of cinnamaldehyde/glycerol (CGA) and 1-naphthaldeyde/glycerol (NGA) (Figure 1). All acetals were tested in the DPPH assay as a screening test to check the potential antioxidant activity. The acetals that presented the best performance were used in preliminary tests of antioxidant resistance with soybean biodiesel.

**Figure 1:**
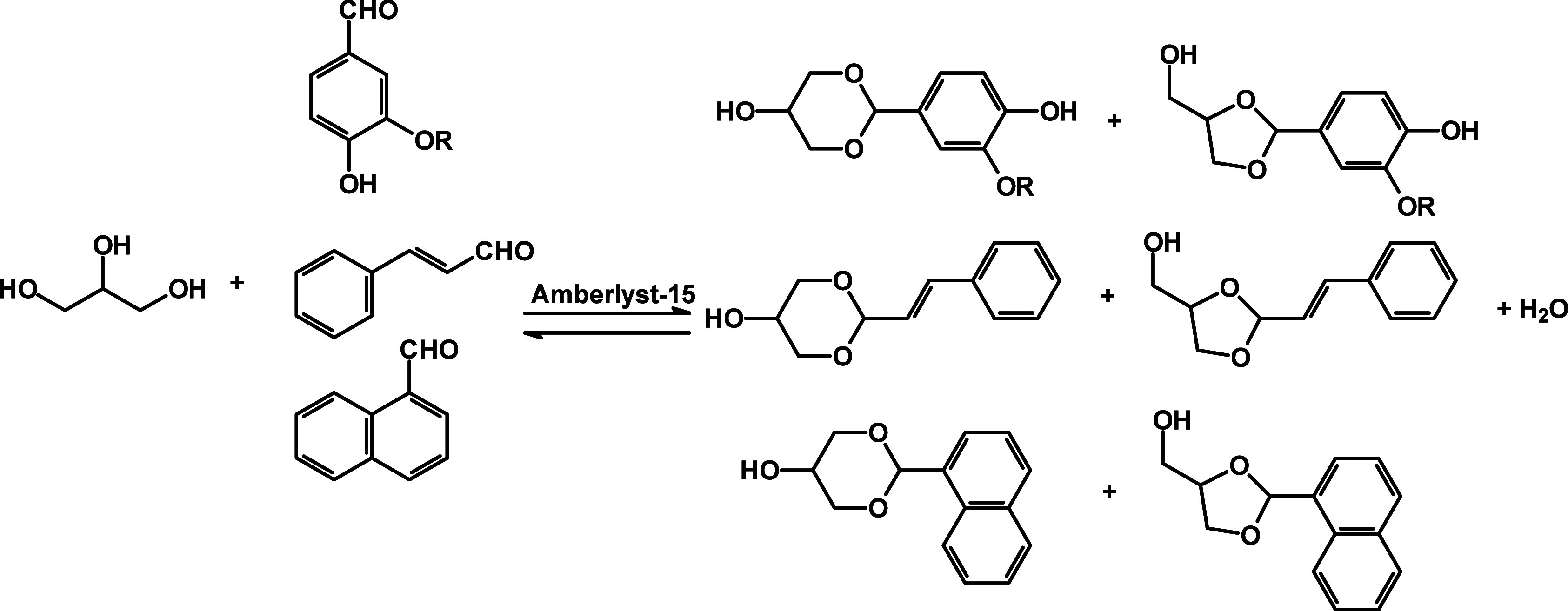
Acid-catalyzed acetalization of glycerol with vanillin (R = methyl), ethyl vanillin (R = ethyl), cinnamaldehyde, and 1-naphthaldehyde.

## Experimental Part

### Materials

Vanillin, ethyl vanillin, cinnamaldehyde, 1-naphthaldehyde, toluene, 2,2-diphenyl-1-picrylhydrazyl (DPPH), and Amberlyst-15 were purchased from Sigma-Aldrich. Sodium bicarbonate, potassium hydroxide, sodium chloride, and anhydrous sodium sulfate were obtained from Vetec (Brazil). Glycerol was purchased from Synth (São Paulo, Brazil). Ethyl acetate, hexane, and all other solvents used were acquired from Isofar (Brazil). All chemicals were of analytical grade and used without further purification.

### Synthesis of the Glycerol Acetals

In a typical procedure, glycerol (130 mmol), Amberlyst-15 (10 wt % relative to the limiting reagent), previously dried at 120 °C for 2 h, and toluene were added to a 100 mL two-neck round-bottom flask. The flask was immersed in a silicone oil heating bath and equipped with a Dean–Stark apparatus for continuous removal of water. The reaction mixture was heated to reflux, after which vanillin or ethyl vanillin (68 mmol) was added. The progress of the reaction was followed by gas chromatography (GC) analyses of samples withdrawn from the medium, pointing out 30 min in the case of vanillin and cinnamaldehyde, 60 min for ethyl vanillin, and 120 min when 1-naphthaldehyde was employed. After completion, the catalyst was removed by vacuum filtration, the liquid phase was washed successively with aqueous sodium bicarbonate solution (3 × 1/3 of the filtrate volume) and water (1 × 1/3 of filtrate volume) to neutralize the medium and remove residual glycerol. The organic phase was concentrated under reduced pressure using a rotary evaporator. The crude product was purified by open-column chromatography on silica gel, using a mixture of hexane and ethyl acetate as the mobile phase to obtain the corresponding glycerol-derived acetals.

### Characterization of the Products

The progress of the reaction was monitored by GC using an Agilent 7890 gas chromatograph. Before each analysis, aliquots containing 50 μL of the reaction mixture were diluted in 1.0 mL of ethyl acetate. Samples (0.2 μL) were injected into an Agilent HP-5MS capillary column of 30 m × 0.25 mm with a 0.25 μm (5%-phenyl)-methylpolysiloxane stationary phase, using helium as carrier gas. The oven temperature was programmed from 120 to 220 °C at a heating rate of 10 °C min^–1^ and held at 220 °C for 2 min.

The compounds were identified by GC–mass spectrometry (MS) using an Agilent 5975C XL MSD quadrupole mass spectrometer with electron impact ionization at 70 eV. The reaction products were also characterized by ^1^H nuclear magnetic resonance (NMR) and ^13^C NMR spectroscopy. Spectra were acquired at 298 K on a Bruker Magneto Ascend 600 Console Avance III HD spectrometer. Samples were prepared by dissolving the isolated products in 0.6 mL of deuterated chloroform (CDCl_3_), and chemical shifts were reported relative to tetramethylsilane as the internal reference.

### DPPH Radical Scavenging Assay

The antioxidant activity was evaluated using the 2,2-diphenyl-1-picrylhydrazyl (DPPH) radical scavenging assay. Spectrophotometric measurements of DPPH absorbance were carried out using an Agilent Cary 60 UV–vis spectrometer. A methanolic solution of DPPH (2.7 mL, 40 μg mL^–1^) was mixed with 0.3 mL of acetal solutions at different concentrations. The mixtures were maintained under controlled conditions, and the absorbance at 515 nm was monitored for up to 60 min. The absorbance of the DPPH radical solution without any added antioxidant was recorded as a control experiment for each experiment. The DPPH radical exhibits a strong absorption band at 515 nm, showing an intense violet color. Upon reaction with the potential antioxidant, the DPPH radicals abstract a hydrogen atom to afford the diphenyl-picryl-hydrazine, which results in the decrease of the absorbance at 515 nm and gradual discoloration of the solution toward pale yellow.^42^

The effective concentration (EC_50_) was determined from absorbance values after 60 min, following adaptation of previously published methods.^43,44^ EC_50_ corresponds to the amount of antioxidant required to reduce the initial DPPH radical concentration by 50%. Lower EC_50_ values indicate a higher antioxidant efficiency. The detailed procedure and the standard calibration curves (Figures S1–S8) are shown in the Supporting Information.

### Rancimat Measurements

The oxidative stability of EVGA and VGA acetals were also tested in a soybean biodiesel formulation, to EN 14112 standard (rancimat test) using a Metrohm 873 Biodiesel Rancimat instrument. For each test, 3.0 g of the biodiesel blended with 1.0 wt % (10,000 ppm) of the acetal additive was placed in the reaction vessel and heated to 110 °C under a continuous flow of filtered compressed air at 10 L h^–1^. This range of antioxidant additive concentration is commonly adopted by Brazilian biodiesel producers. All measurements were performed in triplicate, and the reported induction periods correspond to mean values (standard deviations also included).

The soybean biodiesel was produced from refined soybean oil upon standard transesterification with excess methanol at 65 °C with NaOH as a catalyst. The progress of the reaction was monitored by a previously developed high-performance liquid chromatography method.^45^ The final biodiesel presented 99% of methyl esters.

### Theoretical Calculations

Density functional theory (DFT) calculations were used to assess the bond dissociation energy (BDE). The M062X functional with the def2-TZV basis set was used with the aid of the ORCA package.^46^ This hybrid *meta*-GGA functional was selected because of its reliable performance in calculating thermochemistry and radical reactions.

Frequency calculations were performed at the same level of theory to confirm that all optimized structures correspond to true minima (no imaginary frequencies) and to obtain zero-point energy (ZPE) corrections. Bond dissociation energies were computed according to

$$BDE=E\left(R^{\cdot }\right)+E\left(H^{\cdot }\right)-E\left(R-H\right)$$

with inclusion of ZPE corrections.

For vanillin and ethyl vanillin, two possible hydrogen abstraction sites were investigated: (i) the phenolic O–H bond, (ii) the aldehydic C–H bond. For VGA and EVGA, in addition to the phenolic O–H bond, the methine C–H bond of the acetal moiety was also examined, given its potential to form a resonance-stabilized radical.

## Results and Discussion

### Product Distribution and Characterization of the Acetals

The glycerol acetals were synthesized via an acid-catalyzed reaction using Amberlyst-15. This catalyst has been previously shown to be one of the most active among several heterogeneous materials catalyzing glycerol acetalization and ketalization.^20^ Therefore, we did not explore other heterogeneous catalysts nor carried out deactivation tests in this study as it was not the main purpose. In a previous work,^21^ we have studied the continuous flow ketalization of glycerol with Amberlyst-15 catalyst and found that it is highly active and stable.

A mixture of 2-(4-hydroxy-3-methoxyphenyl)-1,3-dioxan-5-ol and 2-(4-hydroxy-3-methoxyphenyl)-1,3-dioxolane-4-methanol isomers is produced upon the reaction of glycerol with vanillin and will be designated as VGA. The reaction of glycerol with ethyl vanillin produces 2-(3-ethoxy-4-hydroxyphenyl)-1,3-dioxan-5-ol and 2-(3-ethoxy-4-hydroxyphenyl)-1,3-dioxalane-4-methanol isomers, which will be designated as EVGA. A mixture of 2-(2-phenylethenyl)-1,3-dioxan-5-ol and 2-(2-phenylethenyl)-1,3-dioxolane-4-methanol isomers was obtained for the reaction of glycerol with cinnamaldehyde and designated as CGA, whereas reaction of glycerol with 1-naphthaldehyde affords 2-(1-naphthalenyl)-1,3-dioxan-5-ol and 2-(1-naphthalenyl)-1,3-dioxolane-4-methanol acetal isomers, which were named as NGA. No attempt was made to separate the acetal isomers, including the corresponding *Z* and *E* isomers, which were further tested as a mixture. Since we do not intend to exploit them in biological systems, we felt it was unnecessary to proceed with laborious separation and purification procedures to get the antioxidant activity of the pure isomers. Figures S9–S27 of the Supporting Information show the chromatogram and respective mass spectra of the glycerol acetals after purification highlighting the mixture of isomers. The isolated yield was 83% for VGA, 81% for EVGA, 64% for CGA, and 86% for NGA. Usually, the six-membered-ring glycerol acetal is thermodynamically more stable. For instance, the equilibrium distribution for the acetals of glycerol and formaldehyde is 70 and 30% for the six- and five-membered rings, respectively.^47^

The production of vanillin-glycerol (VGA), ethyl vanillin-glycerol (EVGA), and CGA acetals has been previously reported in the literature,^48,49^ but there was no published characterization of these products, which were mostly produced in the context of catalytic activity studies, without being isolated from the reaction mixture. Therefore, we carried out complementary ^1^H and ^13^C NMR structural characterization, which are shown in Figures S28–S43.

The ^1^H NMR spectra of EVGA, VGA, CGA, and NGA show similar features: characteristic signals appear between 4.5 and 3.0 ppm and are attributed to methine, methylene, and hydroxyl protons of the dioxacyclic rings, occurring at lower chemical shifts than the aromatic protons (8.5–6.5 ppm). Four single signals between 6.6 and 4.9 ppm are assigned to the acetal protons of the *Z* and *E* isomers of the five- and six-membered rings acetals. In the same region, phenolic proton signals for EVGA and VGA are also observed, as well as the characteristic doublet signals of benzylic hydrogen atom in CGA, due to the presence of one hydrogen in its vicinity. The presence of the double bond in CGA is also highlighted between 7.0 and 6.0 ppm due to the chemical shifts of the hydrogen atoms bonded to the olefinic sp^2^ carbon atoms.

The ^13^C NMR spectra display signals for all carbon atoms of the acetals, with particular emphasis on the sp^3^ acetal carbon atom (benzylic carbon). DEPT-90 confirmed the presence of carbons bonded to a single hydrogen, including sp^3^ carbons bonded to −OH and −CH_2_OH groups (60–80 ppm), benzylic sp^3^ carbon atoms, characteristic of each isomer (100–110 ppm), and aromatic sp^2^ carbons. For the latter, assignments between 110 and 120 ppm were observed for EVGA and VGA, whereas NGA and CGA showed signals at slightly higher shifts (120–140 ppm), supporting the structural assignment of the synthesized acetals. DEPT-135 analyses, in addition to confirming the DEPT-90 assignments, highlights signals for the methyl carbon atoms of the ethoxide group (10–20 ppm) in EVGA and the methylene carbons of the dioxacyclic five- and six-membered rings, as well as their substituents (−CH_2_OH)—the latter observed only in the five-membered ring (60–80 ppm).

### Antioxidant Activity (DPPH Assay)

The antioxidant performance, quantified by their EC_50_ values obtained from the DPPH radical scavenging assay, is summarized in Table 1. Lower EC_50_ values correspond to a higher radical scavenging efficiency.

**Table 1:** Antioxidant Activity Measured by the DPPH Assay^d^

sample	EC_50_ ^a^	*R* ^2^ ^b^	reference
EVGA	2.1	0.974	this work
VGA	5.5	0.999	this work
AGA^c^	80.0	-	^37^
CGA	54.7	0.949	this work
NGA	38.3	0.947	this work
BHT	1.0	0.901	this work
vanillin	21.5	0.932	this work
ethyl vanillin	32.6	0.986	this work

a Expressed in mol of the sample per mol of DPPH.

b Correlation coefficient of the DPPH + target oxidant experiments (see the Supporting Information for details).

c AGA stands for anisaldehyde–glycerol acetal.

d (EC_50_ expressed as mol of the sample per mol of DPPH).

The ethyl vanillin-glycerol acetal (EVGA) showed an EC_50_ value of 2.1 mol of the sample per mol of DPPH, whereas the VGA presented an EC_50_ of 5.5 mol of the sample per mol of DPPH, confirming their strong radical scavenging capability. Notably, the antioxidant efficiencies of EVGA and VGA approach that of BHT, a conventional antioxidant widely used in fuel, cosmetic, and food applications, which showed an EC_50_ of 1.0 mol of the sample per mol of DPPH under the same test conditions. BHT is derived from fossil resources and has been associated with environmental and toxicological concerns. On the other hand, EVGA and VGA are completely derived from renewable feedstocks, making them promising candidates for applications as green and efficient antioxidants.

In a previous study,^37^ we have reported the antioxidant activity of AGA. Nevertheless, the EC_50_ of AGA is significantly higher (Table 1) than the values found for EVGA, VGA, and NGA, stressing the superior performance of these acetals as antioxidants. CGA showed EC_50_ lower than AGA but significantly higher than the other acetals of this study, indicating a moderate to poor performance as antioxidant in the DDPH assay. Thus, the extension of the conjugated π system in CGA was not sufficient to greatly stabilize the radical formed upon abstraction of the methine hydrogen atom.

EVA and VGA presented higher antioxidant activity in the DPPH assay than NGA, indicating that the presence of phenolic groups in the acetals plays a more important role in the stabilization of the formed radical than the extended conjugated π system in NGA. In fact, the radical formed upon abstraction of the methine hydrogen atoms of NGA is not well stabilized because the aromaticity is lost in several resonance structures. In contrast, the presence of an *ortho*-methoxy group provides additional stabilization for the radical formed upon the abstraction of the phenolic hydrogen atom in EGVA and VGA, leading to lower EC_50_. As a result, NGA presented inferior antioxidant activity than EVGA and VGA but superior activity with respect to AGA and CGA.

In fact, EVGA and VGA showed improved antioxidant activity when compared to ethyl vanillin and vanillin precursors, which showed EC_50_ values of 32.6 and 21.5, respectively. Vanillin acetals have been used as sensory stimulants for use in cosmetics, foods, and beverages.^50^ For instance, the acetal formed upon the reaction of ethyl vanillin and propylene glycol (EVPG) finds applications as a fragrance in cosmetics and electronic cigarettes.^51^ The toxicity of EVPG has been evaluated in rats.^52^ Above 300 mg kg^–1^ daily dosage, the product can cause liver problems. To the best of our knowledge, there is no report of antioxidant properties of vanillin and ethyl vanillin acetals, especially those produced from the reaction with glycerol. Therefore, EVGA and VGA have the potential to be explored as antioxidants in food, beverage, cosmetics, and other sectors.

Comparison of different potential antioxidant molecules is not straightforward,^53^ as the EC_50_ is not universal and may vary with the methodology and range of concentrations used. For instance, the reported EC_50_ of BHT in the DPPH assay spans from 3.45 to 31.5 mg mL^–1^.^54^ In this work, we measured and expressed the EC_50_ of BHT as 1.0 mol per mol of DPPH. Thus, the methodology and form of expressing the results may give different insights, making an absolute comparison among target antioxidant molecules very difficult. The comparison is valid when a series of potential antioxidant molecules are tested under the same conditions in a particular test, as was the case for this study. Therefore, we cannot make reliable comparisons of EVGA and VGA to other renewable or natural antioxidants.

In addition to the issues pointed out in the previous paragraph, many articles report the antioxidant activity of solvent extracts containing several natural products rather than a pure compound.^55,56^ However, there is a study^57^ on the antioxidant activity of vanillin and ethyl vanillin in the DPPH assay, although the EC_50_ was not reported, the overall reactivity was not significantly lower when compared, for instance, to that of vanillyl alcohol. This result stresses the effect of substituting an aldehyde moiety with a CH_2_OH group, similar to what was carried out in the present study, which transformed the aldehydes into acetals.

### Rancimat Tests with Biodiesel

EVGA and VGA were selected for preliminary tests of antioxidant resistance with soybean biodiesel together with AGA for comparison purposes. Besides showing the best antioxidant performance in the DDPH assay, they can be considered green because of their synthesis from renewable, biomass-based raw materials. We did not consider using CGA in these tests because of the poor antioxidant potential, whereas NGA cannot be considered green as the synthesis of 1-naphthaldehyde usually involves hazardous chemicals and nonrenewable feedstocks.^58^

Table 2 shows the results of the EN 14112 standard test (rancimat). One can see that EVGA and VGA increased the induction period in the rancimat test relative to the standard soybean biodiesel without any antioxidant additive. Although the values are not outstanding, they stress the antioxidant potential of these two glycerol acetals in a practical application. On the other hand, adding the same proportion of AGA to the soybean biodiesel did not lead to a significant increase in the induction period, indicating its poor antioxidant activity as expressed in the DDPH assay. Therefore, the initial working hypothesis of including a phenolic OH in the glycerol acetal seems to be a rational approach. Besides improving the antioxidant activity in the DPPH assay, it also led to improved performance in a practical application on the use of glycerol acetals as an antioxidant in biodiesel formulations.

**Table 2:** Induction Period Measured According to EN 14112 Standards for Soybean Biodiesel Samples with 10,000 ppm of the Acetals

antioxidant	induction period (h)	standard deviation (h)
none	2.71	0.19
EVGA	4.53	0.22
VGA	3.33	0.06
AGA	2.81	0.23

### Theoretical Calculations

The results of the DPPH assay indicated that the acetalization of vanillin and ethyl vanillin produces acetals with improved antioxidant activity. To better understand the experimental results and try to establish a possible structure–activity relationship, we carried out DFT calculations of the BDE of selected O–H and C–H bonds (Figure 2). The BDE gives an indication of the stability of the radical formed, which may be associated with the antioxidant activity of the compounds. We stress that calculations were not carried out with the aim of understanding the mechanism of the reaction but rather to try to offer some feasible explanation for the increased antioxidant activity of EVGA and VGA relative to ethyl vanillin and vanillin.

**Figure 2:**
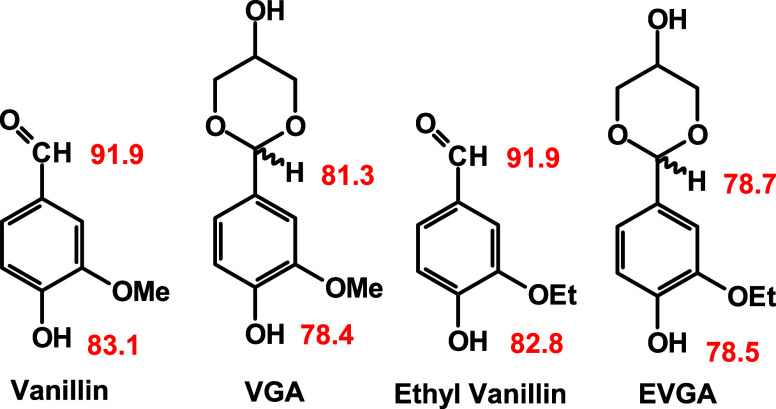
Calculated BDE. Values in kcal mol^–1^.

For vanillin and ethyl vanillin, two possibilities were investigated: cleavage of the phenolic O–H bond and cleavage of the C–H bond of the aldehyde group. As expected, the BDE of the phenolic O–H functionality is significantly lower than the aldehydic C–H, in agreement with literature results at other levels of calculations, that indicates a BDE within 79–87 kcal mol^–1^ for the phenolic OH bond and within 84 –91 kcal mol^–1^ for the aldehydic C–H bond.^59^ Therefore, the antioxidant activity of vanillin and ethyl vanillin is mostly associated with the abstraction of the phenolic hydrogen atom and formation of a delocalized free radical. Upon acetalization with glycerol, the BDE of the phenolic OH decreases from 83.1 to 78.4 kcal mol^–1^ in the case of VGA, whereas for EVGA, the BDE goes from 82.8 to 78.5 kcal mol^–1^. The results support the experimental findings and can be explained by the better electron-donating ability of the *p*-CHO_2_ moiety (acetal functionalization) in VGA and EVGA, in contrast to the electron-withdrawing ability of the *p*-CHO group of vanillin and ethyl vanillin. From linear free-energy relationship, the observed σ of an acetal group (*p*-CHO_2_) is around +0.1,^60^ indicative of an almost neutral electronic effect on the ring. By contrast, the aldehyde group is electron-withdrawing, with σ of +0.43 (*p*-CHO). Thus, on EVGA and VGA acetals, the radical formed upon the abstraction of the phenolic hydrogen atom is more stabilized than the radical formed upon the abstraction of the same hydrogen atom in vanillin and ethyl vanillin, explaining their superior antioxidant activity results.

The calculations also suggest that the methine C–H group (benzylic position) of the acetal moiety in EVGA and VGA might act as antioxidant centers too. The calculated BDE is lower than the values of the phenolic hydrogen atom in vanillin and ethyl vanillin and of similar magnitude of the BDE of the phenolic OH moiety in EVGA and VGA. Thus, these acetals may have two potential antioxidant centers, although we cannot indicate which one is predominant from the present experiments. The abstraction of the methine hydrogen atom of EVGA and VGA forms a well-delocalized radical, which may explain the lower computed BDE values (Figure 3). We stress again that this work was not aimed at studying the complete reaction scheme of the antioxidant activity of the glycerol acetals but rather offered a potential explanation for their improved antioxidant activity.

**Figure 3:**
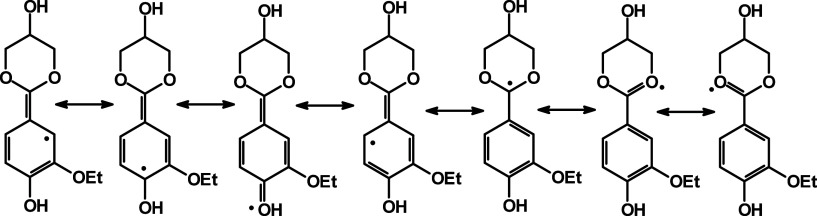
Resonance structures of the radical formed upon the abstraction of the methine hydrogen atom of EVGA.

## Conclusions

The reaction of glycerol with vanillin, ethyl vanillin, cinnamaldehyde, and 1-naphthaldehyde was successfully carried out in the presence of Amberlyst-15 acid catalyst. The mixture of the acetal isomers, named EVGA, VGA, CGA, and NGA for the reaction with ethyl vanillin, vanillin, cinnamaldehyde, and 1-naphthaldehyde, respectively, was properly isolated from the reaction medium and characterized by GC/MS and NMR.

The antioxidant performance of the isolated acetals was tested through a DPPH radical scavenging assay. EVGA and VGA showed significantly improved antioxidant activity than the other glycerol acetals and also relative to ethyl vanillin and vanillin precursors, respectively, with performance close to that of BHT, a traditional fossil-based antioxidant. They also presented significantly improved performance compared to AGA, previously reported to present antioxidant activity in the DPPH assay.

EVGA and VGA acetal isomers were added to soybean biodiesel and showed an increased induction period in the racimat test relative to the neat biodiesel sample, without any added antioxidant, confirming the potential of both glycerol acetals as green antioxidants, produced from renewable feedstocks.

The explanation of the superior performance of EVGA and VGA may be related to the lower BDE as computed by DFT calculations. The presence of the acetal group in the para position relative to the phenolic OH decreases the BDE, affording a more stable free radical. The BDE of the methine hydrogen atom of the acetal group was similar in magnitude to the BDE of the phenolic hydrogen atom, implying that EVGA and VGA may have two potential antioxidant centers.

The acetalization of vanillin and ethyl vanillin with glycerol produces a mixture of acetal isomers with potential use as efficient antioxidants for multiple purposes, being a potential green substitute for BHT.

## Supplementary Material



## Notes

Detailed procedure for obtaining the EC_50_, together with the standard calibration curve for each experiment, CG, mass spectrum, and ^1^H and ^13^C NMR of the acetals (PDF)
