# Supplementary material for: Glycerol-Vanillin Acetals as Green and Efficient Antioxidants
Source: ACS Omega. 2026 Jun 12;11(25):38160–7. doi: 10.1021/acsomega.6c03930 (PMC13325385; doi:10.1021/acsomega.6c03930)
Supplement: Supplementary file 1 [file ao6c03930_si_001.pdf]

Supplementary Information

Glycerol-Vanillin Acetals as Green and Efficient Antioxidants

Daniella R. Fernandes,[a] Júlio C. G. de Almeida,[a] Nilton Rosenbach Jr,[b]

Claudio J. A. Mota [a,c]*

[a] Universidade Federal do Rio de Janeiro, Instituto de Química. Av. Athos da Silveira Ramos 149, CT Bl A, 21941-909, Rio de Janeiro, Brazil.

[b] Faculdade de Ciência Exatas e Engenharias, Universidade do Estado do Rio de Janeiro, Rio de Janeiro 23070-200, Brazil.

[c] Universidade Federal do Rio de Janeiro, Escola de Química. Av. Athos da Silveira Ramos 149, CT Bl E, 21941-909, Rio de Janeiro, Brazil.

*e-mail: cmota@iq.ufrj.br

Experimental procedure for determining the EC_{50}

1) *Preparation of the DPPH solution*

2.0 mg (2000 mg) of DPPH was solubilized in methanol, and the volume was adjusted to 50.00 mL. The solution must be stored at 4 ^{o}C (refrigerator) and in the dark for further use.

2) *Standard calibration curve for DPPH*

The previous prepared solution was used to prepare 10.00 mL methanol solutions of varied DPPH concentrations (0, 1, 5, 10, 15, 20, 25, 30, 35 e 40 $\mu g.mL^{-1}$). After 1h in the dark, a sample of 3 mL of each solution was transferred to a cuvette, and the absorbance was measured at 515 nm. Pure methanol was used as a blank. New calibration curves were determined for each experiment with the antioxidant samples to have better accuracy. Figure S1 shows one of the standard calibration curves measured for the methanolic DPPH solution, which can be expressed by equation 1. Only curves with a correlation coefficient (R^2) above 0.900 were considered.

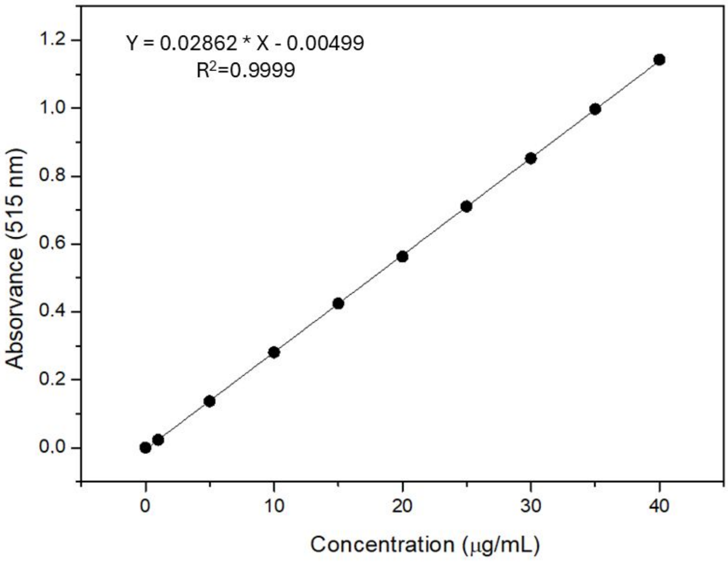

Figure S1: Standard calibration curve for the DPPH methanolic solution taken just previously the measurement of the DPPH + EVGA experiment.

$$Y_1 = 0.02862\ X_1 - 0.00499 \qquad R^2 = 0.9999 \qquad \text{Eq. 1}$$

X_1 – DPPH concentration;

Y_1 – Absorbance at 515 nm;

R^2 – Correlation coefficient

3) *Determination of the antioxidant activity EC_{50}*

5 mg (5000 µg) of the target antioxidant (vanillin, ethyl vanillin, VGA, EVGA, and BHT) was solubilized in methanol and the volume was adjusted to 10.00 mL. The solutions were stored in the dark at -20 ^{o}C.

From the previous prepared solutions, 5.00 mL methanolic solutions were prepared at varied concentrations of the target antioxidant (25, 50, 100, 150, 200, and 250 µg.mL^{-1}). For each concentration and target antioxidant, a sample of 0.3 mL (300 µL) was transferred to a cuvette in the dark, and then 2.7 mL of the DPPH solution previously prepared (40 µg.mL^{-1}) was added.

The efficiency concentration (EC_{50}) was taken from the absorbance at 60 min (complete stabilization of the system) for each concentration of the target antioxidant studied. A graph is plotted expressing the variation of the DPPH concentration with the concentration of the target antioxidant. From the linear regression of the graph, an equation for each target antioxidant can be taken, expressing the antioxidant activity of the sample. New calibration curves for the DPPH solution were taken for each experiment to improve accuracy. Only the linear regressions with correlation coefficients (R^2) above 0.9000 were considered. The graphs for each DDPH + target oxidant are shown in Figures S2 to S8.

The detailed steps of calculation for EVGA are shown below; the same general method was used for the other tested antioxidant, using a DPPH calibration curve taken just prior to the experiments.

1) Initial absorbance of the control DPPH solution = 1.025

2) Half of the initial absorbance of the control DPPH solution **=** 0.513

3) Calculus of half the amount of the DPPH to be reacted using eq 1:

$0.513 = 0.02862X_1 - 0.00499 \Leftrightarrow X_1 = 18.10\ \mu g.mL^{-1}$

For 2.7 mL of solution $\Leftrightarrow$ **Z_1 = 48.87 µg of DPPH**

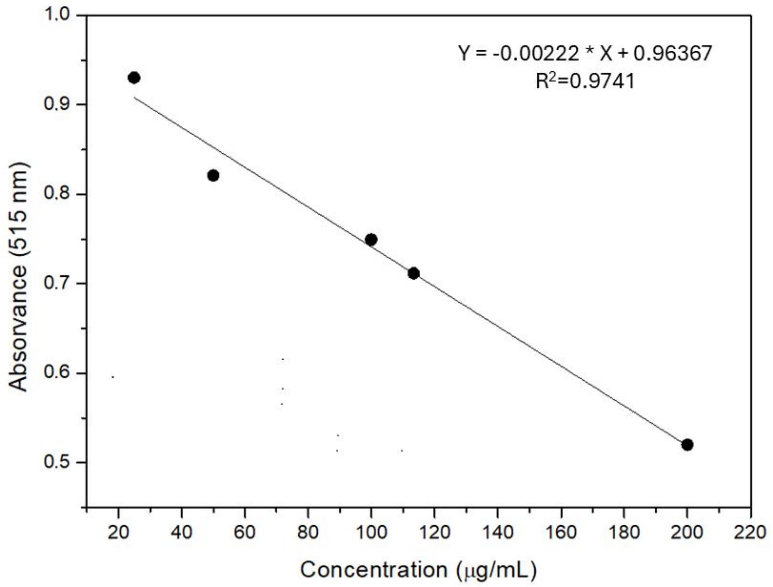

Figure S2: Linear regression of the absorbance after 60 min with the concentration of EVGA in experiments of DPPH decay.

$$Y_2 = -\ 0.00222\ X_2 + 0.96367 \qquad R^2 = 0.9741 \qquad \text{Eq. 2}$$

4) From Eq. 2, one can calculate the amount of the target antioxidant to react with DPPH, considering half of the initial absorbance calculated in step 2, as mass of the target antioxidant per volume of solution:

$0.513 = -\ 0.00222\ X_2 + 0.96367 \Leftrightarrow X_2 = 203.00\ \mu g.mL^{-1}$

For 0.3 mL ⇔ **Z_2 = 60.90 μg of EVGA**

5) The EC_{50} was calculated taken the ratio of Z_2 to Z_1, and will be expressed in mass of the target antioxidant per mass of DPPH:

$Z_2 = 60.90/48.87 = 1.25 \Leftrightarrow EC_{50} = 1.25$ g of EVGA per g of DPPH

6) To convert mol of the target antioxidant to mol of DPPH, it is necessary to divide the obtained mass EC_{50} by the respective mol ratio of the compounds:

Mol of DPPH = 394 g Mol of EVGA = 240 g

1.25.(394/240) ⇔ **EC_{50} = 2.05 mol of EVGA per mol of DPPH**

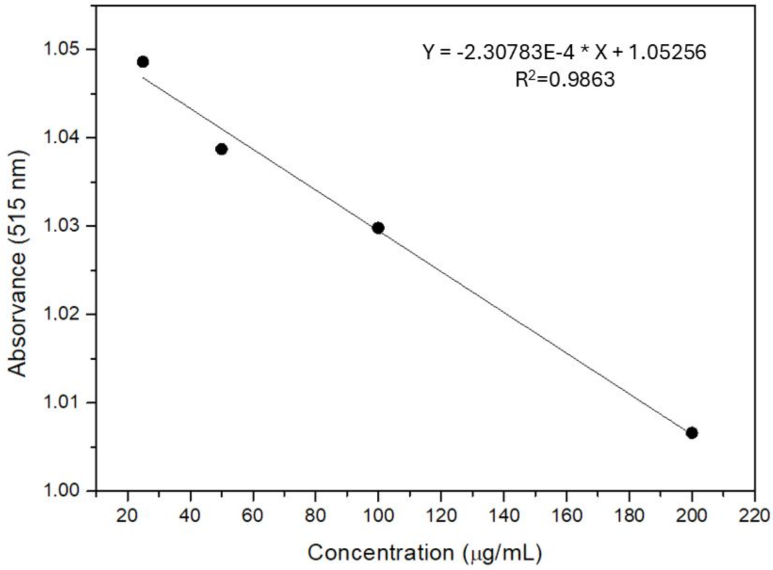

Figure S3: Linear regression of the absorbance after 60 min with the concentration of Ethyl vanillin in experiments of DPPH decay.

$$Y_2 = -\ 2.30783 \times 10^{-4} X_2 + 1.05256 \qquad R^2 = 0.9863$$

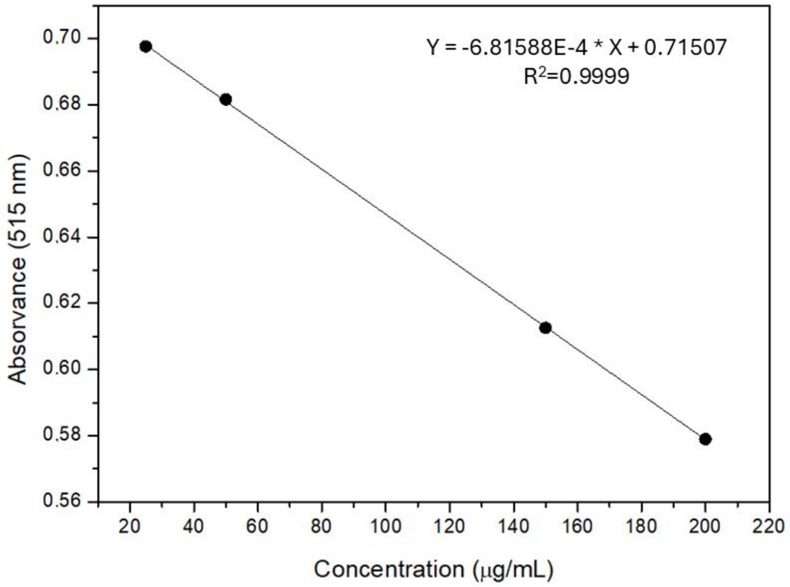

Figure S4: Linear regression of the absorbance after 60 min with the concentration of VGA in experiments of DPPH decay.

$$Y_2 = - 6.81588 \times 10^{-4} X_2 + 0.71507 \qquad R^2 = 0.9998$$

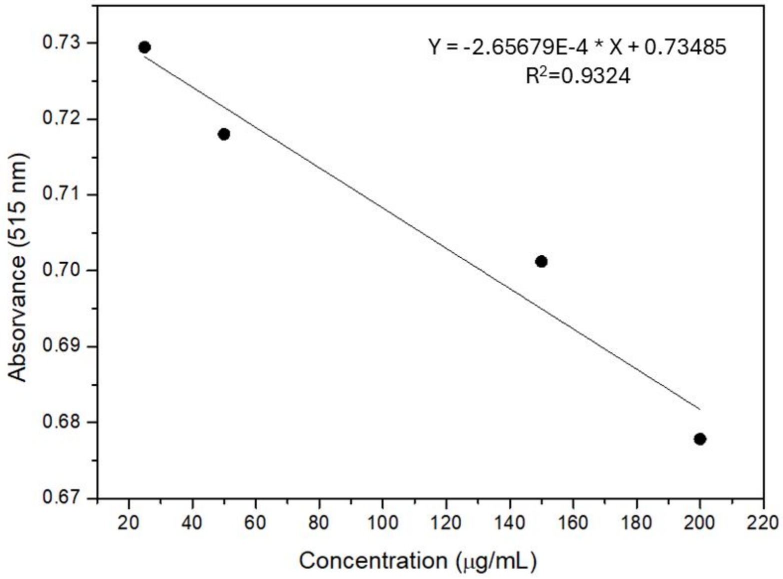

Figure S5: Linear regression of the absorbance after 60 min with the concentration of Vanillin in experiments of DPPH decay.

$$Y_2 = - 2.65679 \times 10^{-4} X_2 + 0.73485 \qquad R^2 = 0.9324$$

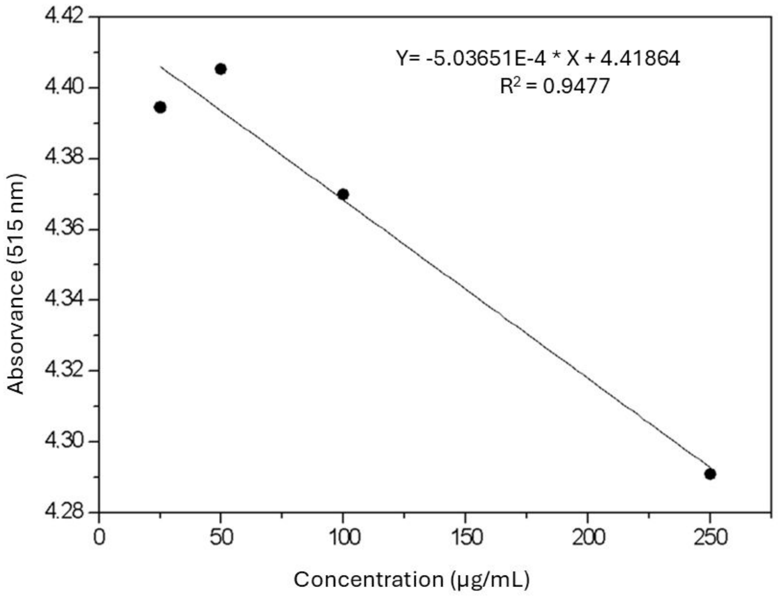

Figure S6: Linear regression of the absorbance after 60 min with the concentration of NGA in experiments of DPPH decay.

$$Y_2 = -5{,}03651 \times 10^{-4} X_2 + 4{,}41864 \qquad R^2 = 0{,}9477$$

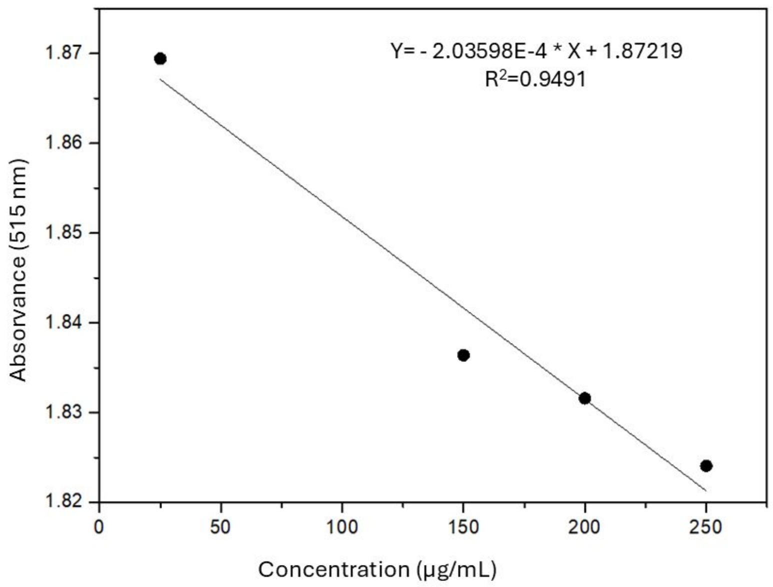

Figure S7: Linear regression of the absorbance after 60 min with the concentration of CGA in experiments of DPPH decay.

$$Y_2 = - 2{,}03598 \times 10^{-4} X_2 + 1{,}87219 \qquad R^2 = 0{,}9491$$

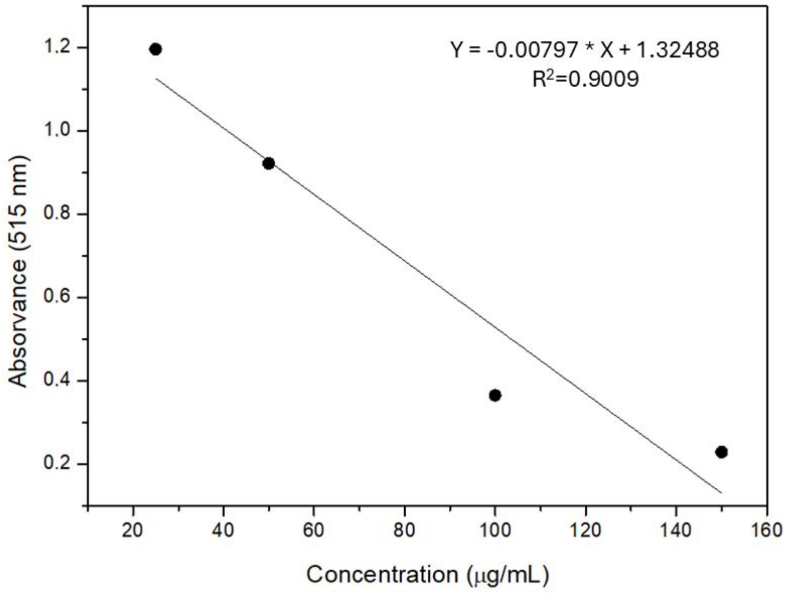

Figure S8: Linear regression of the absorbance after 60 min with the concentration of BHT in experiments of DPPH decay.

$$Y_2 = -\ 0.00797\ X_2 + 1.32488 \qquad R^2 = 0.9009$$

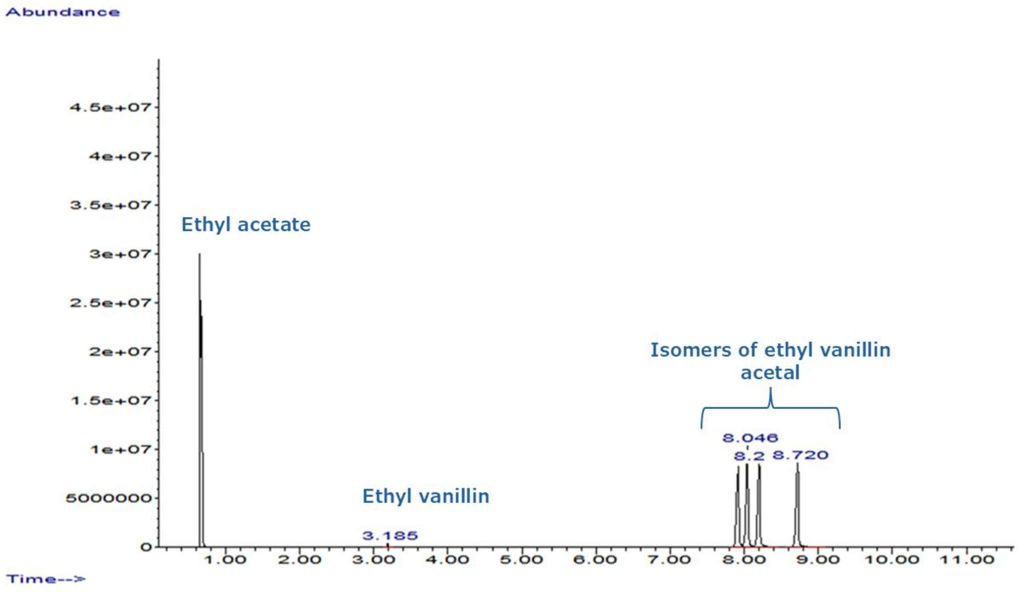

Figure S9: Chromatogram of the acetal mixture obtained from the acetalization of glycerol with ethyl vanillin, highlighting the 4 EVGA isomers.

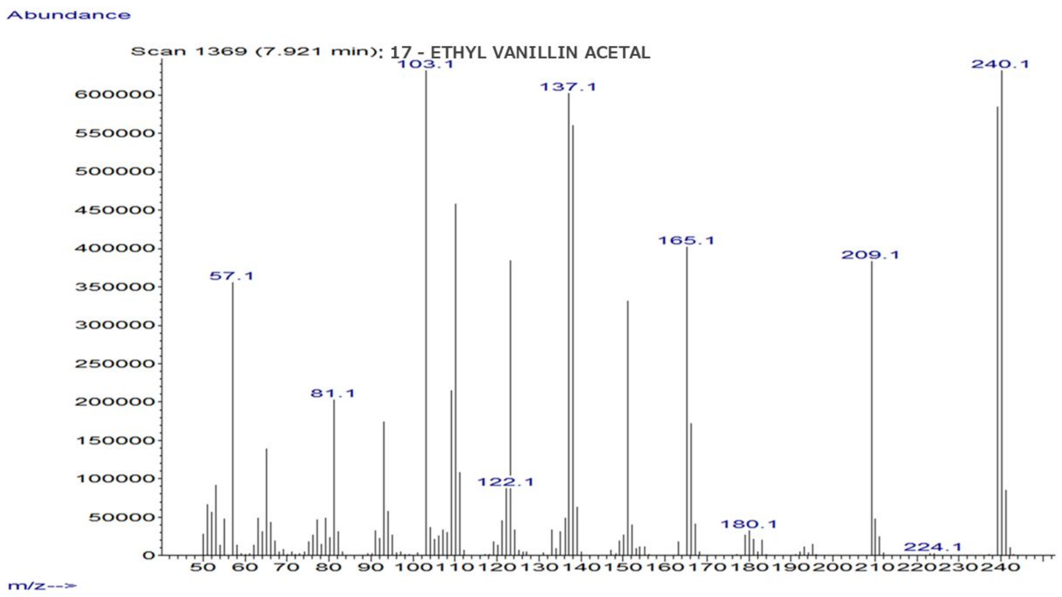

Figure S10: Mass spectrum relative to the EVGA eluting at approximately 7.9 min.

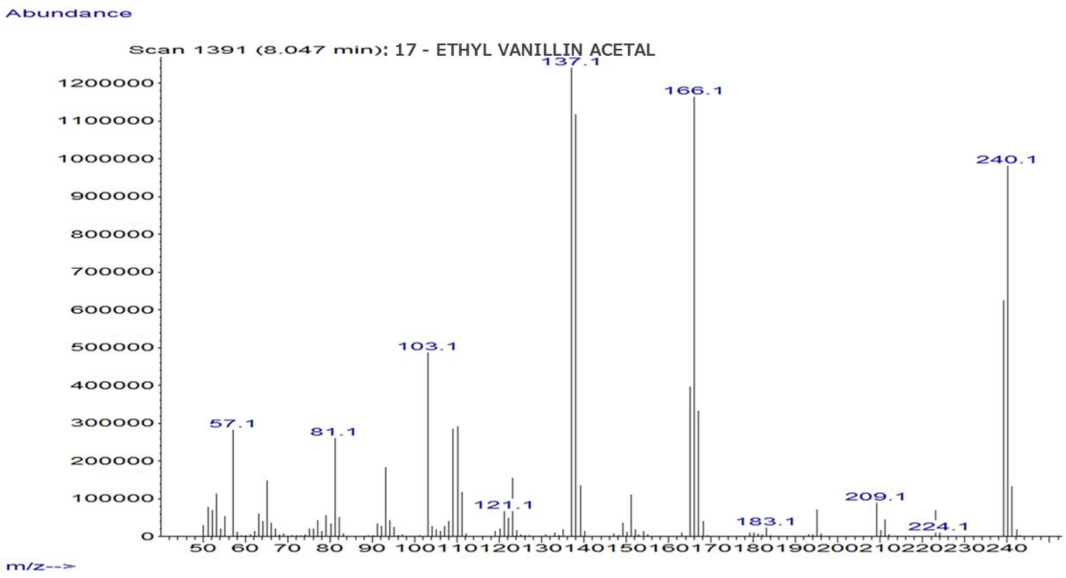

Figure S11: Mass spectrum relative to the EVGA eluting at approximately 8.0 min.

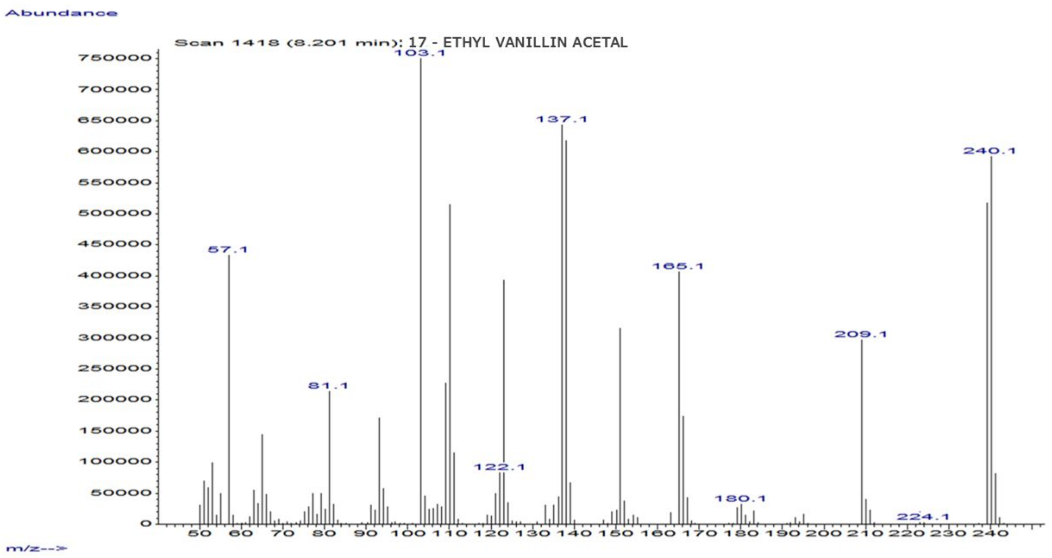

Figure S12: Mass spectrum relative to the EVGA eluting at approximately 8.2 min.

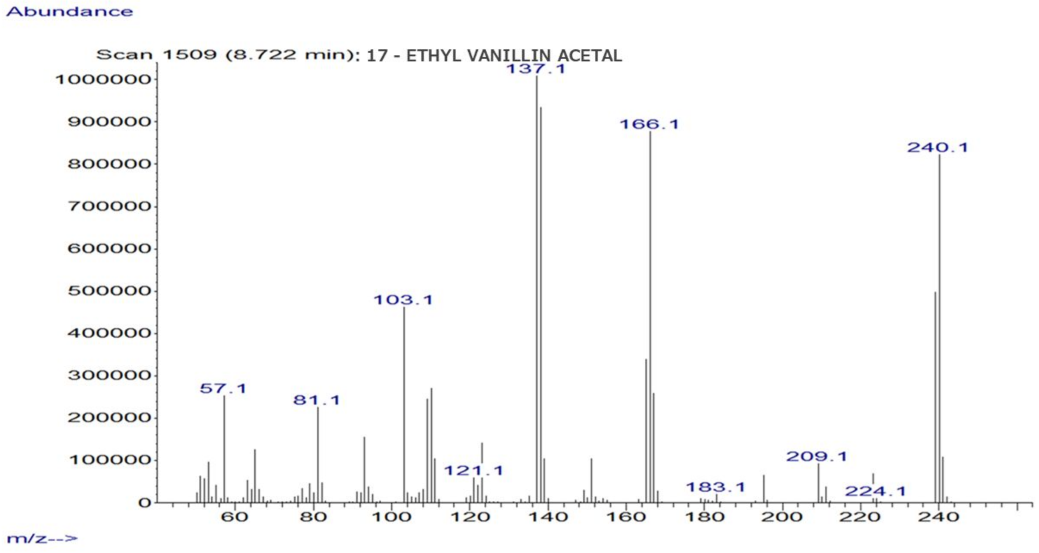

Figure S13: Mass spectrum relative to the EVGA eluting at approximately 8.7 min.

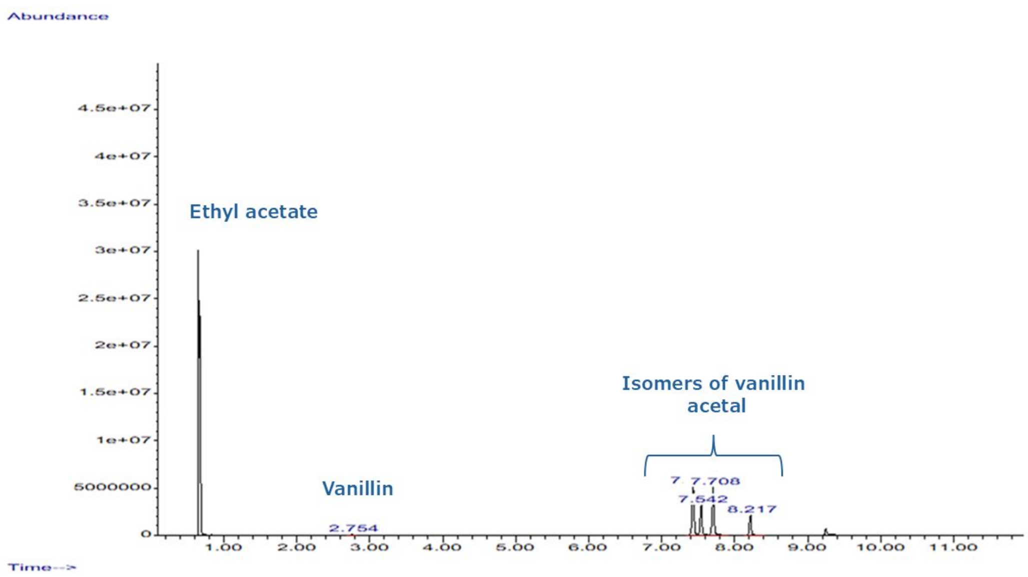

Figure S14: Chromatogram of the acetal mixture obtained from the acetalization of glycerol with vanillin, highlighting the 4 VGA isomers.

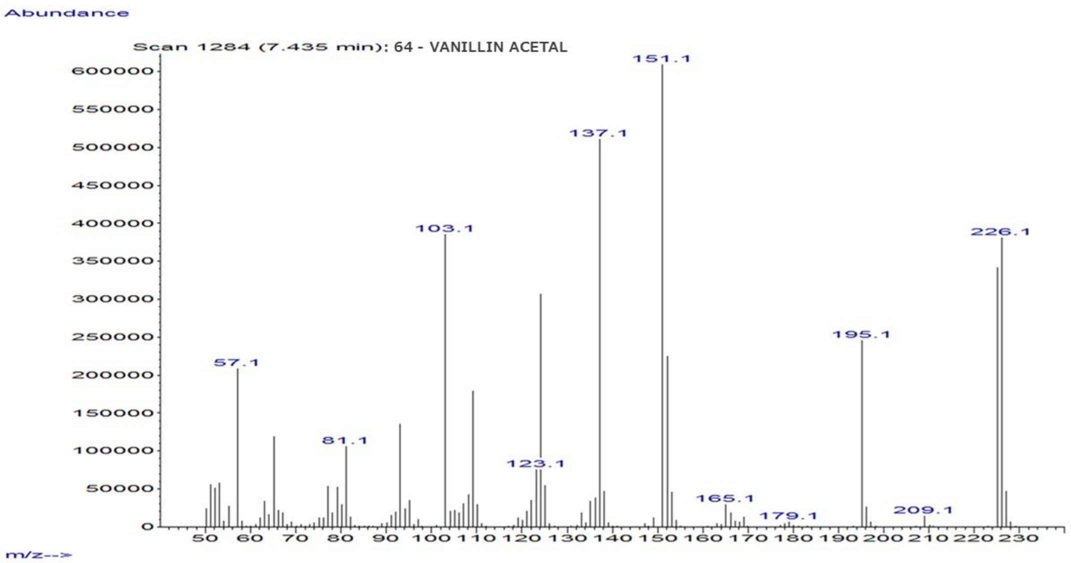

Figure S15: Mass spectrum relative to the VGA eluting at approximately 7.4 min.

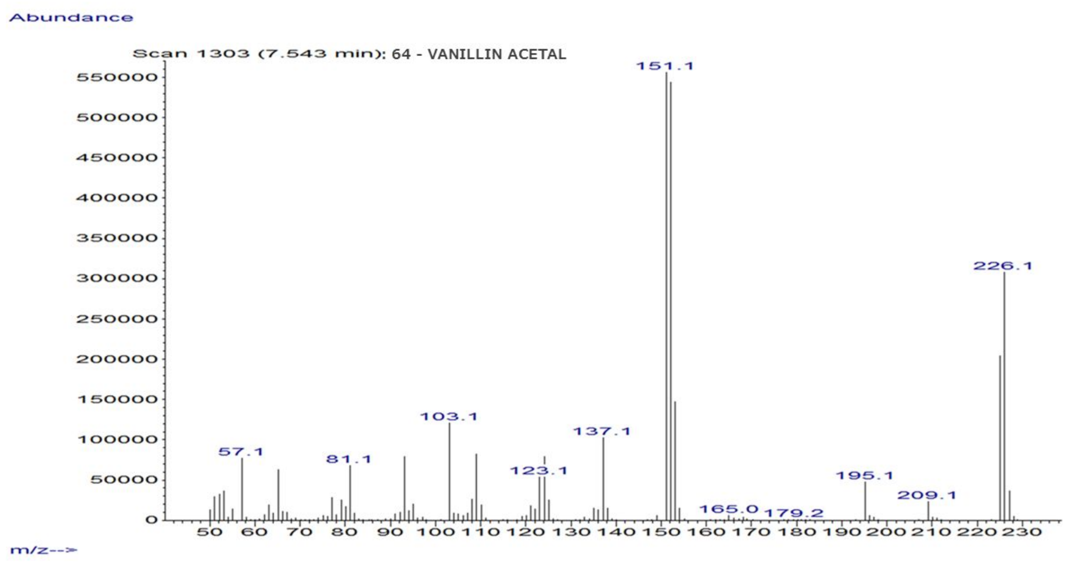

Figure S16: Mass spectrum relative to the VGA eluting at approximately 7.5 min.

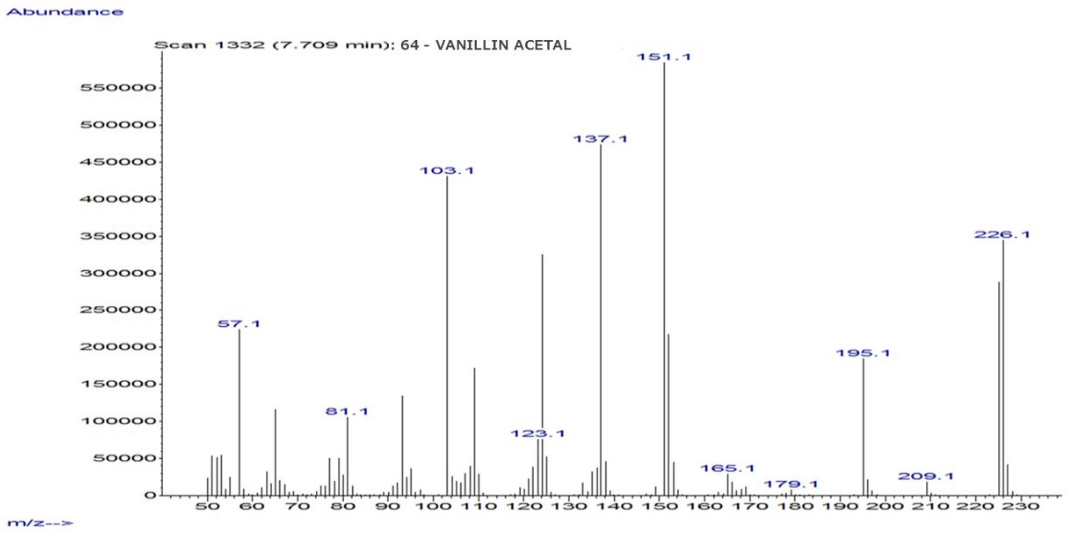

Figure S17: Mass spectrum relative to the VGA eluting at approximately 7.7 min.

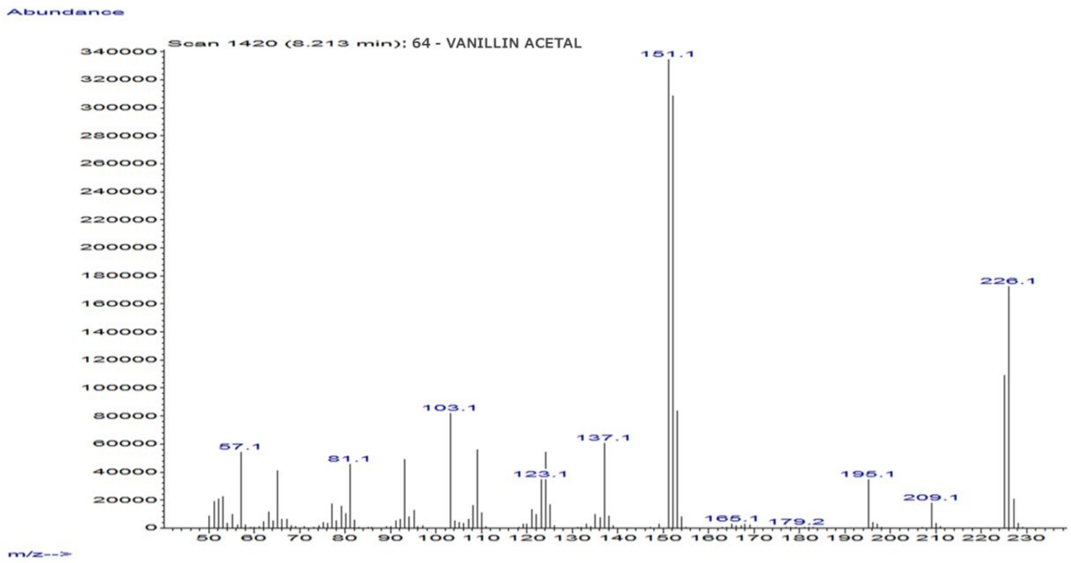

Figure S18: Mass spectrum relative to the VGA eluting at approximately 8.2 min.

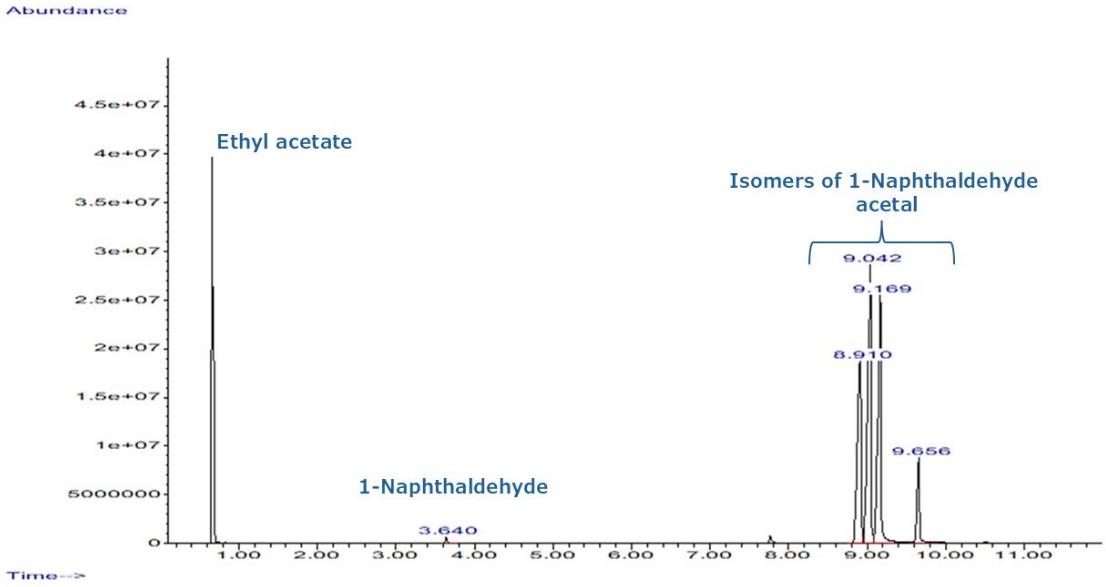

Figure S19: Chromatogram of the acetal mixture obtained from the acetalization of glycerol with 1-naphthaldehyde, highlighting the 4 VGA isomers.

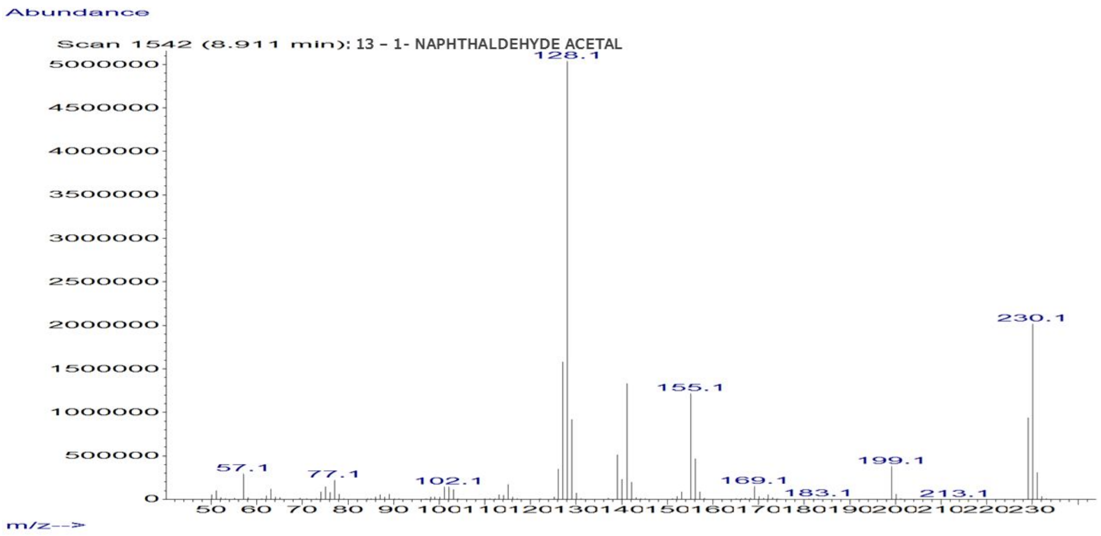

Figure S20: Mass spectrum relative to the NGA eluting at approximately 8.9 min.

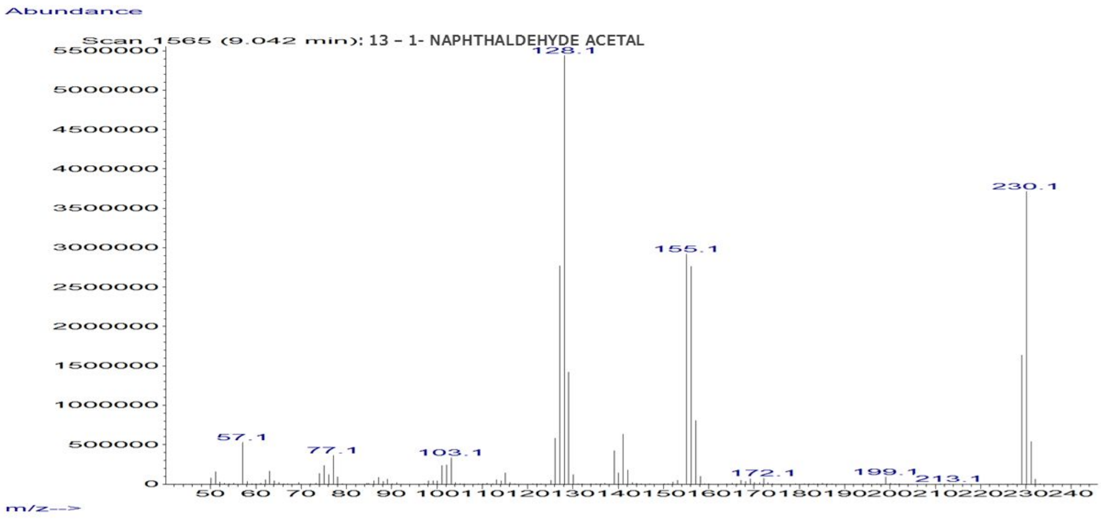

Figure S21: Mass spectrum relative to the NGA eluting at approximately 9.0 min.

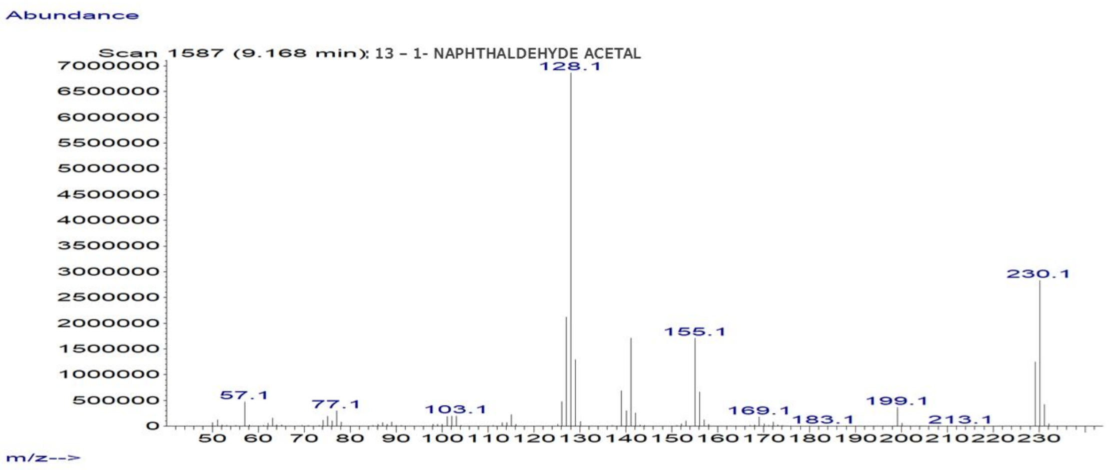

Figure S22: Mass spectrum relative to the NGA eluting at approximately 9.1 min.

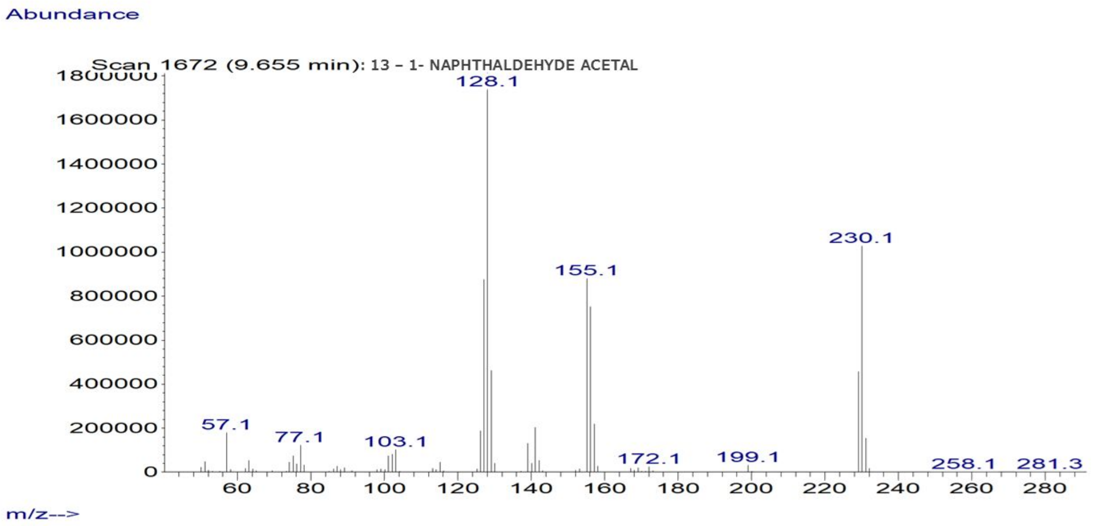

Figure S23: Mass spectrum relative to the NGA eluting at approximately 9.6 min.

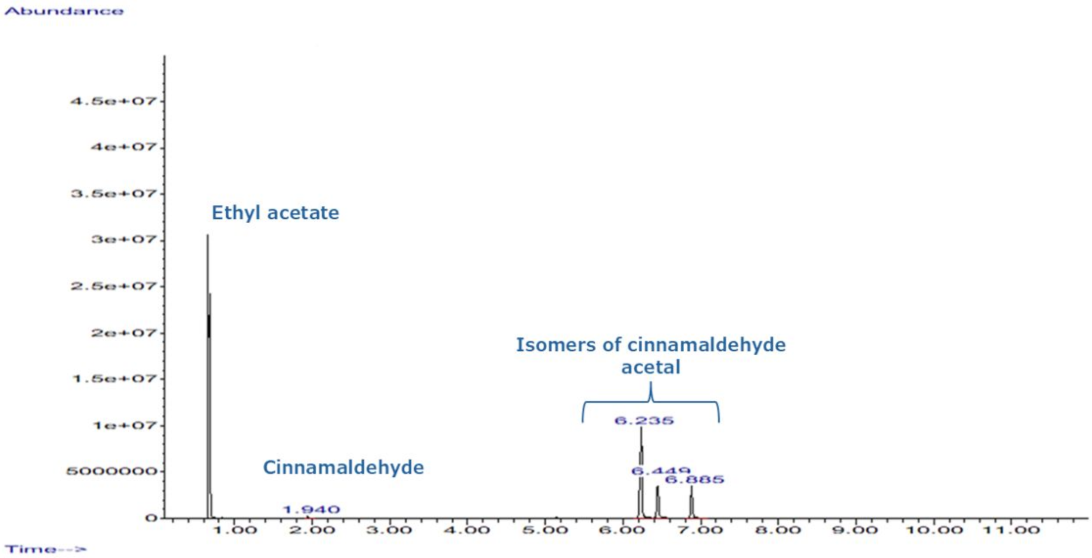

Figure S24: Chromatogram of the acetal mixture obtained from the acetalization of glycerol with cinnamaldehyde, highlighting the 3 VGA isomers.

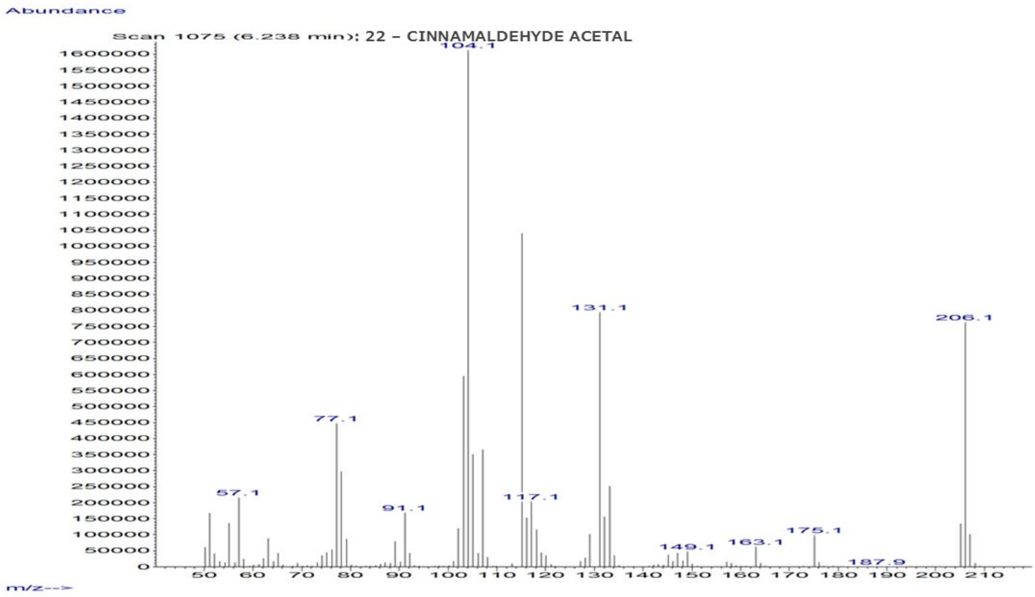

Figure S25: Mass spectrum relative to the CGA eluting at approximately 6.2 min.

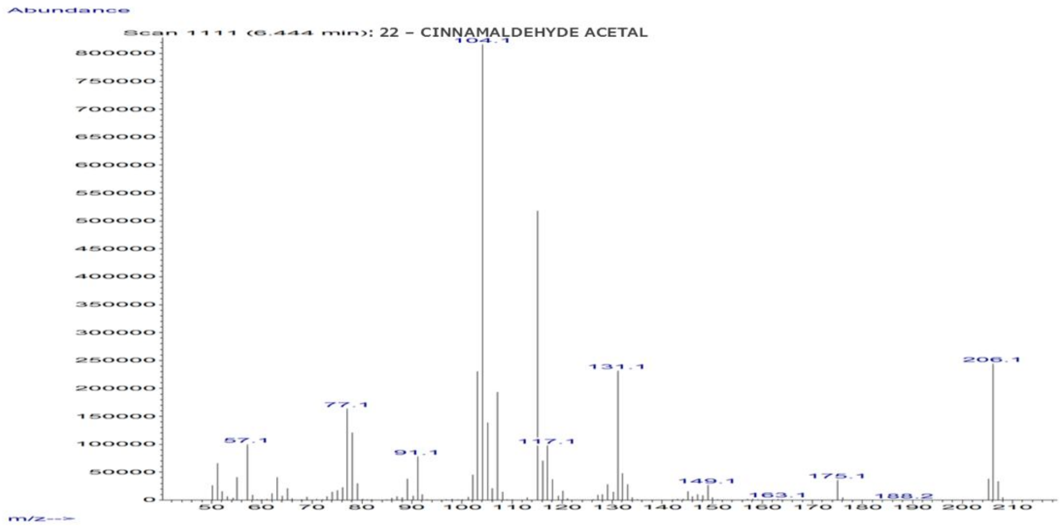

Figure S26: Mass spectrum relative to the CGA eluting at approximately 6.4 min.

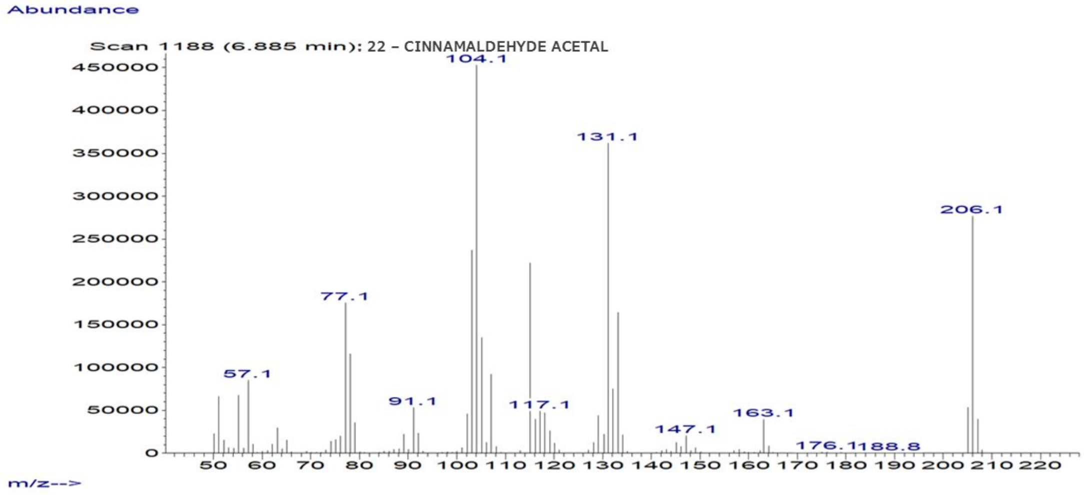

Figure S27: Mass spectrum relative to the CGA eluting at approximately 6.8 min.

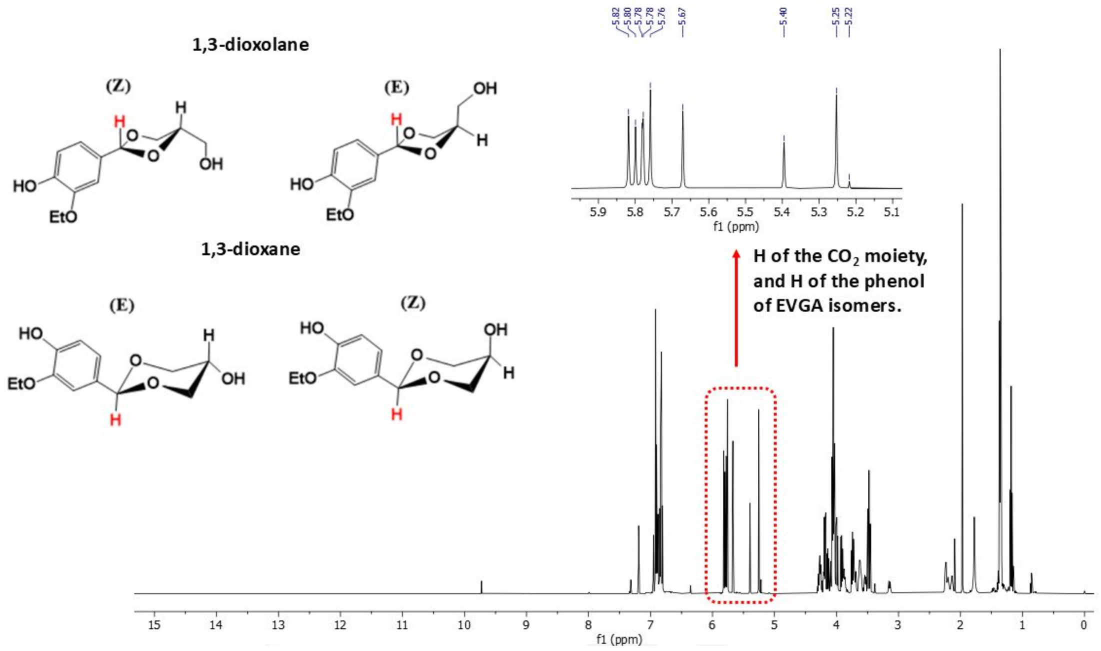

Figure S28: ^{1}H NMR of spectrum of EVGA isomers mixture, isolated from the acetalization reaction of glycerol with ethyl vanillin. R=ethyl.

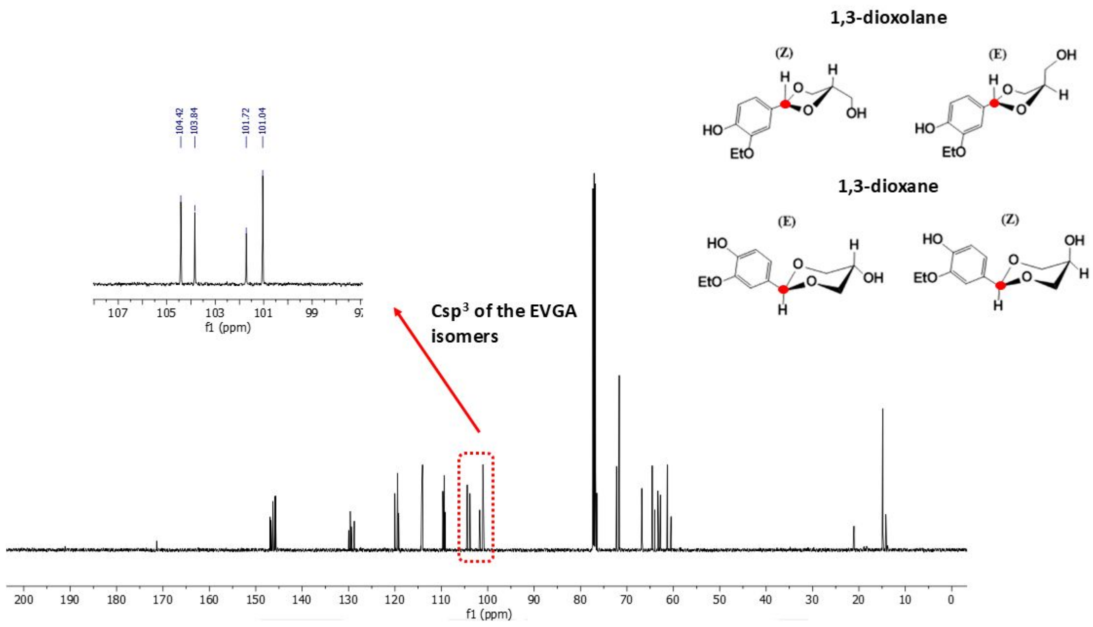

Figure S29: ^{13}C NMR of spectrum of EVGA isomers mixture, isolated from the acetalization reaction of glycerol with ethyl vanillin. R=ethyl.

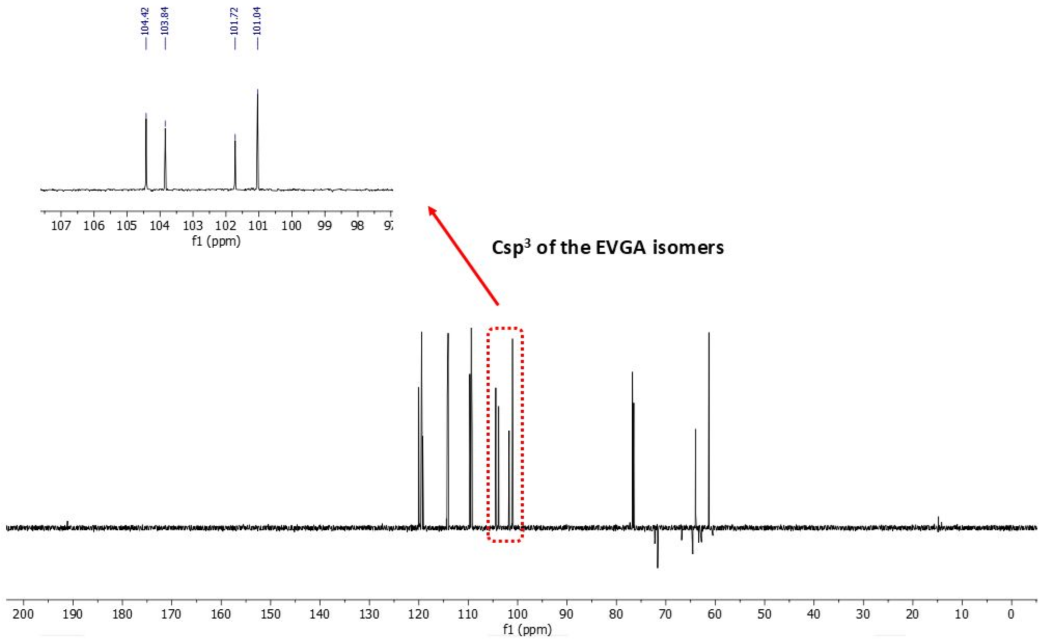

Figure S30: DEPT 90 ^{13}C NMR spectrum of EVGA isomers mixture, isolated from the acetalization reaction of glycerol with ethyl vanillin. R=ethyl.

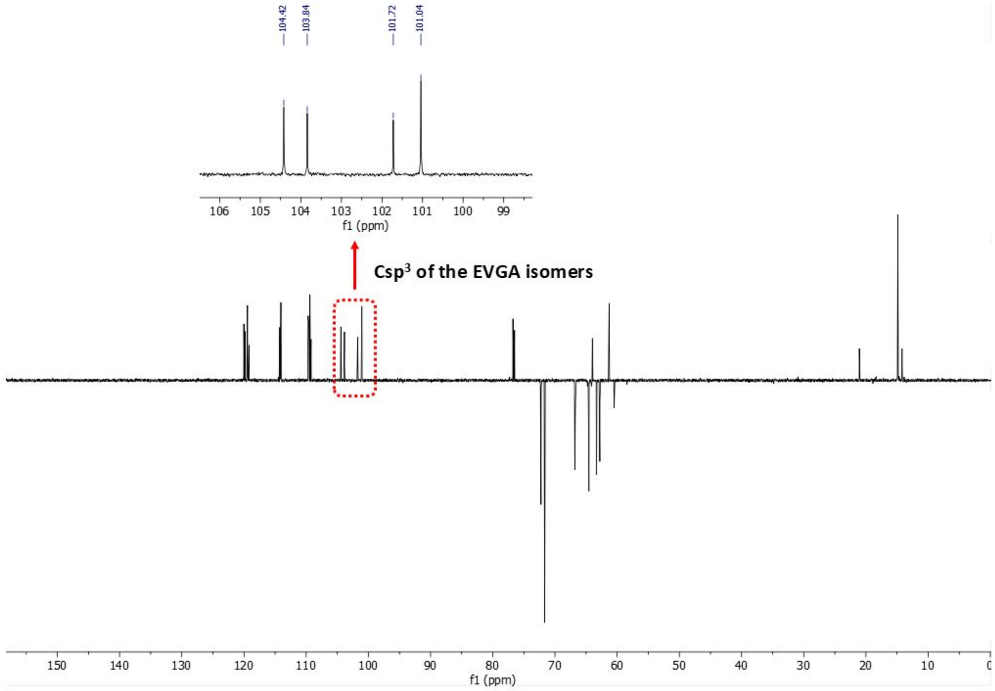

Figure S31: DEPT 135 ^{13}C NMR spectrum of EVGA isomers mixture, isolated from the acetalization reaction of glycerol with ethyl vanillin. R=ethyl.

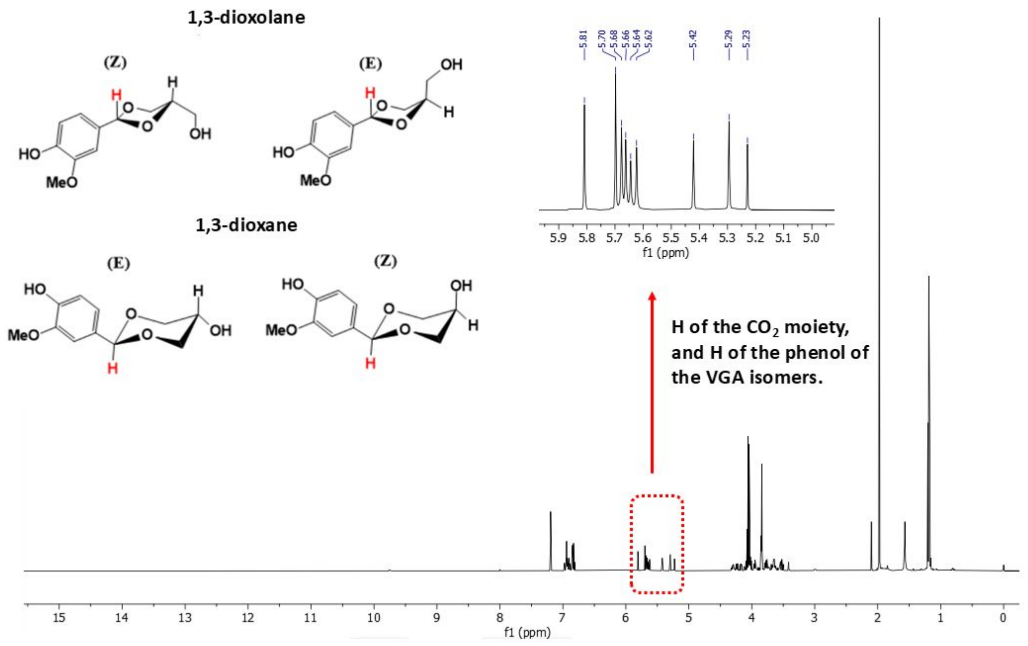

Figure S32: ^{1}H NMR of spectrum of VGA isomers mixture, isolated from the acetalization reaction of glycerol with vanillin. R=methyl.

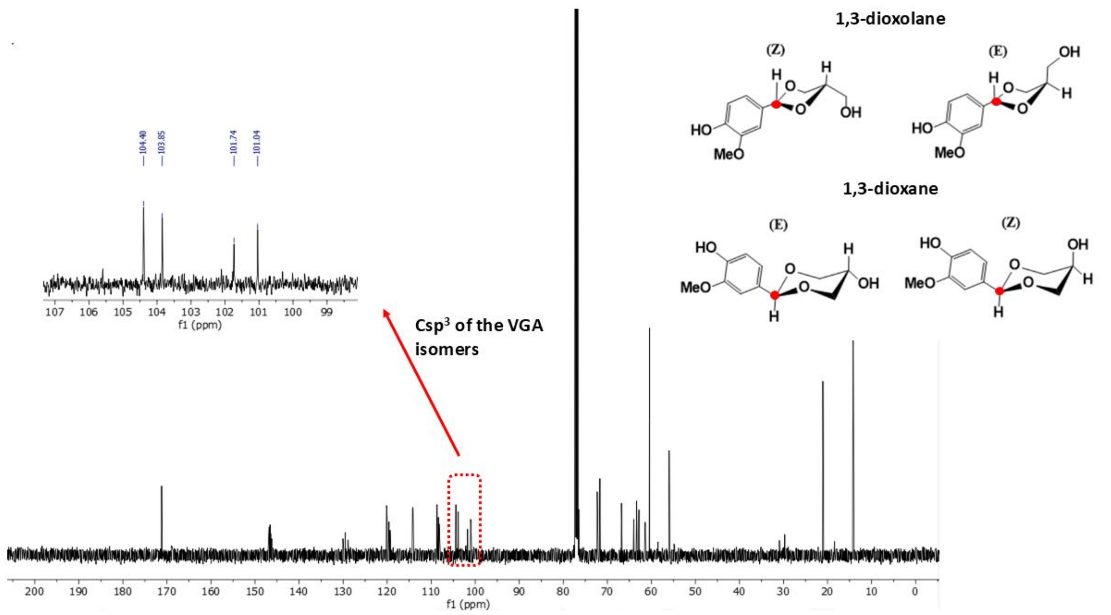

Figure S33: ^{13}C NMR of spectrum of VGA isomers mixture, isolated from the acetalization reaction of glycerol with vanillin. R=methyl.

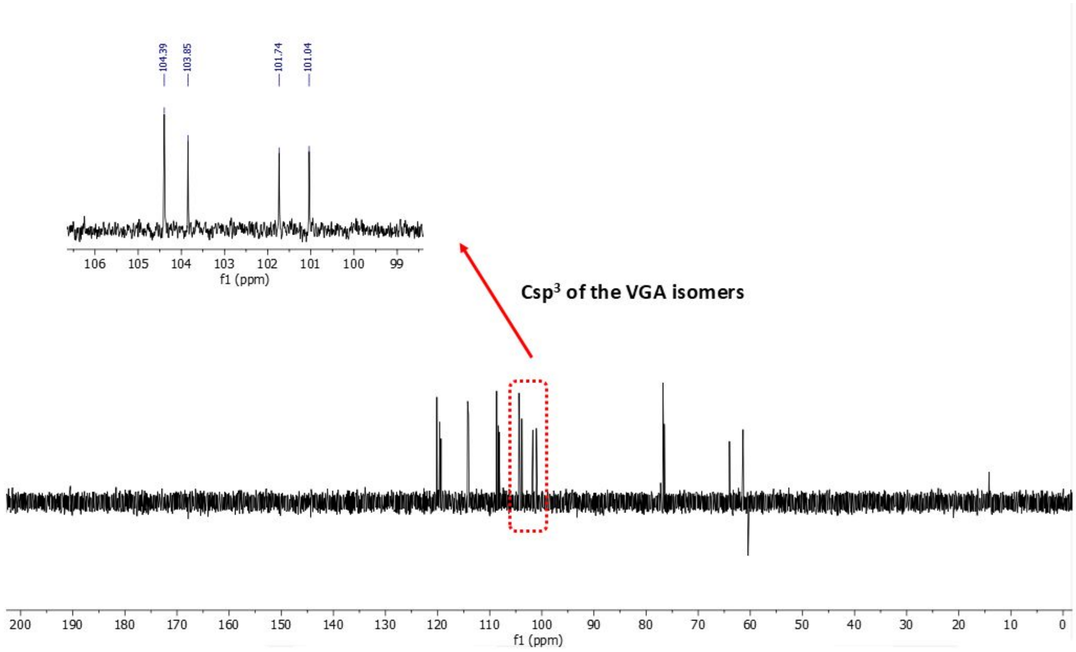

Figure S34: DEPT 90 ^{13}C NMR spectrum of VGA isomers mixture, isolated from the acetalization reaction of glycerol with vanillin. R=methyl.

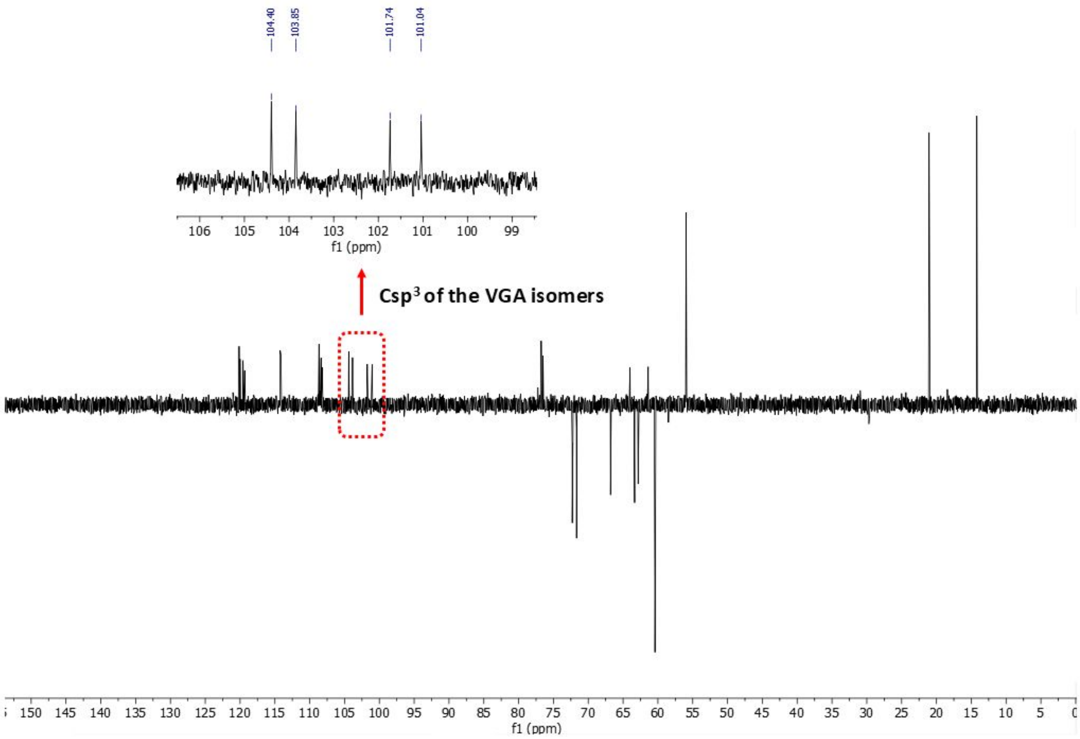

Figure S35: DEPT 135 ^{13}C NMR spectrum of VGA isomers mixture, isolated from the acetalization reaction of glycerol with vanillin. R=methyl.

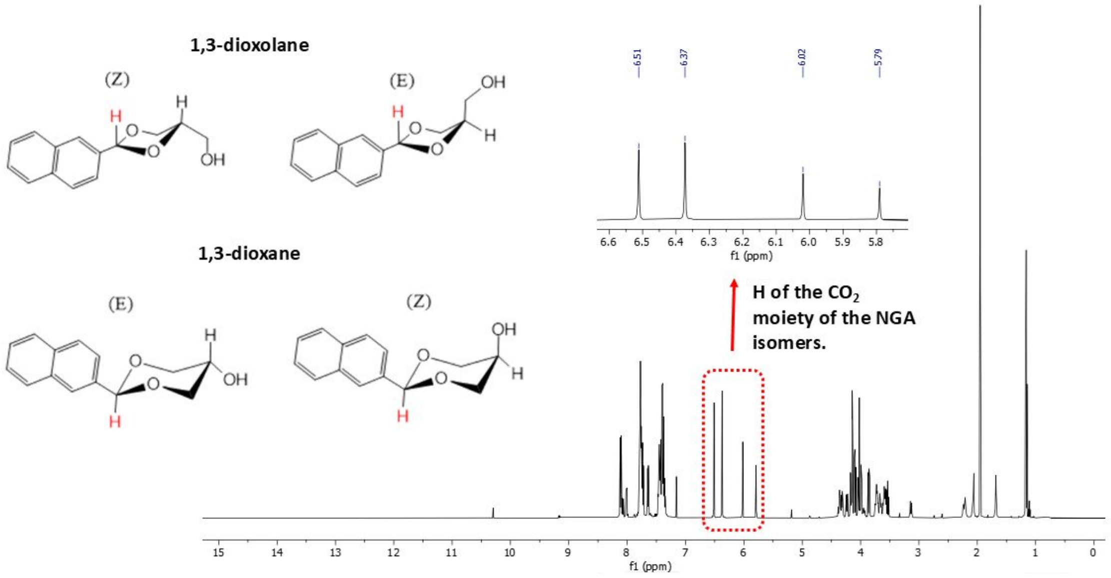

Figure S36: ^{1}H NMR of spectrum of NGA isomers mixture, isolated from the acetalization reaction of glycerol with 1-naphthaldehyde.

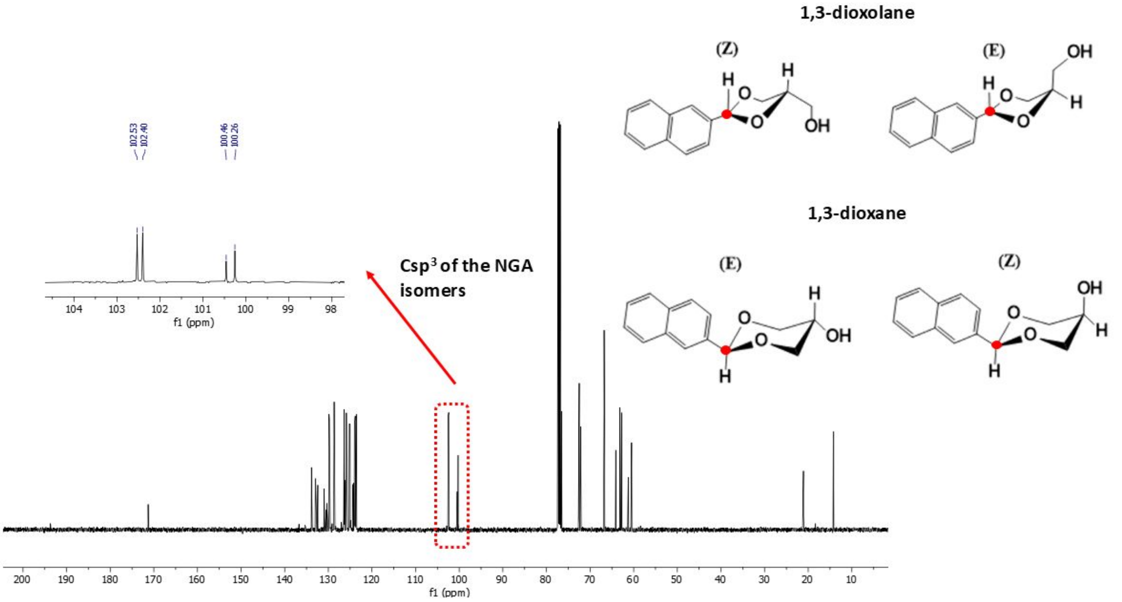

Figure S37: ^{13}C NMR of spectrum of NGA isomers mixture, isolated from the acetalization reaction of glycerol with 1-naphthaldehyde.

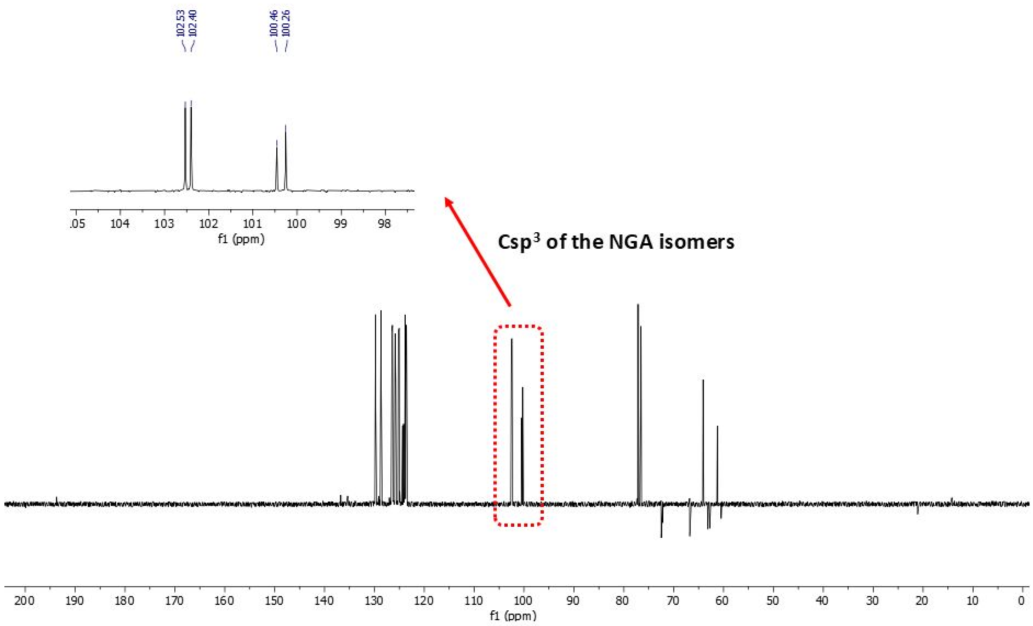

Figure S38: DEPT 90 ^{13}C NMR spectrum of NGA isomers mixture, isolated from the acetalization reaction of glycerol with 1-naphthaldehyde.

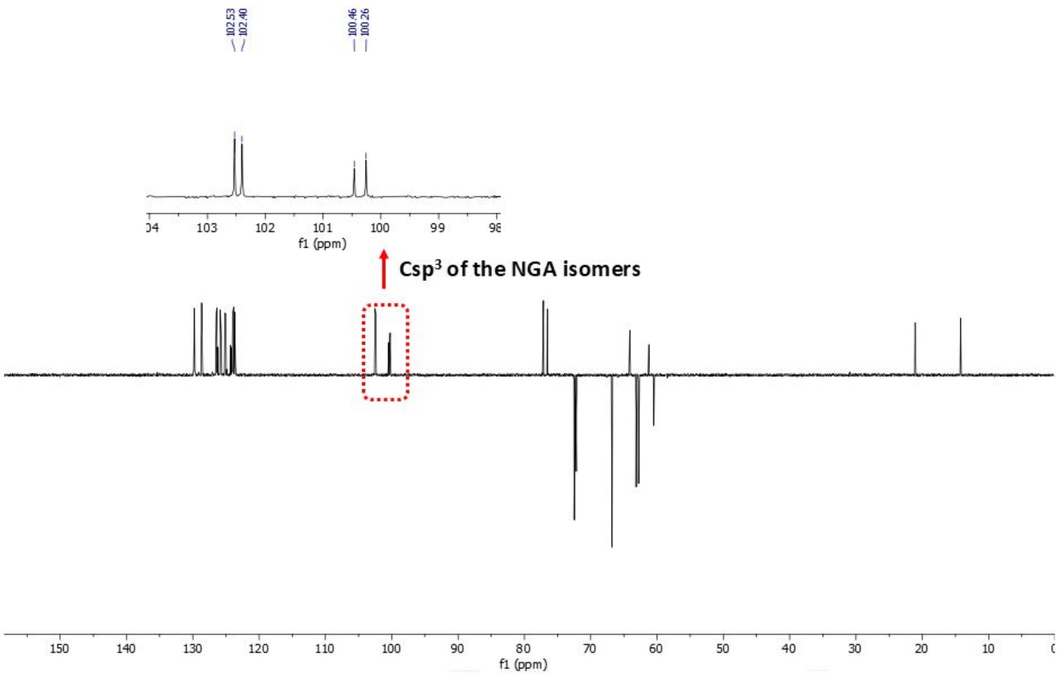

Figure S39: DEPT 135 ^{13}C NMR spectrum of NGA isomers mixture, isolated from the acetalization reaction of glycerol with 1-naphthaldehyde.

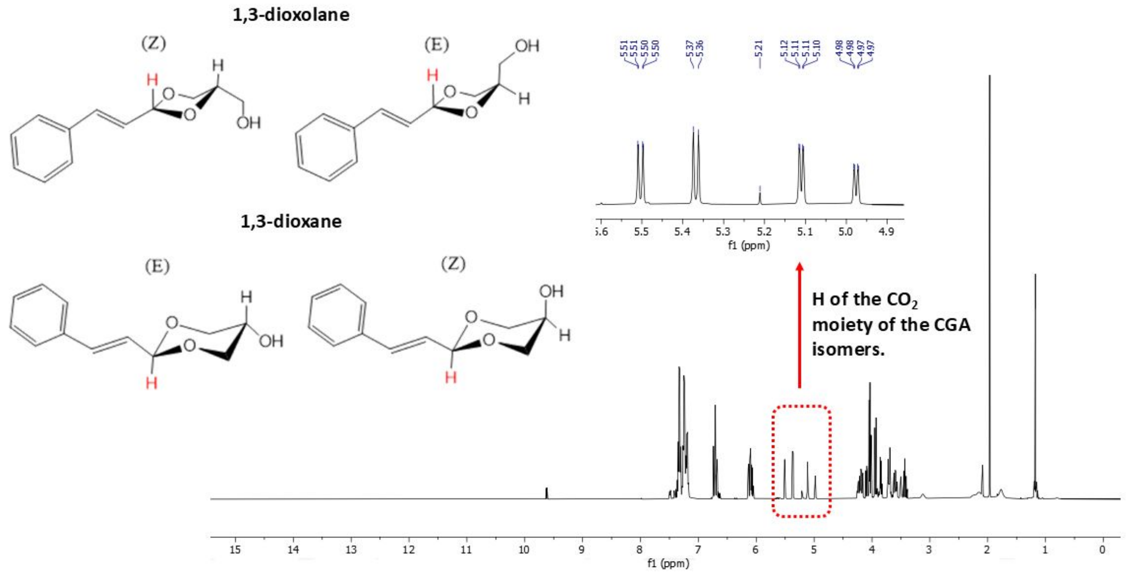

Figure S40: ^{1}H NMR of spectrum of CGA isomers mixture, isolated from the acetalization reaction of glycerol with cinnamaldehyde.

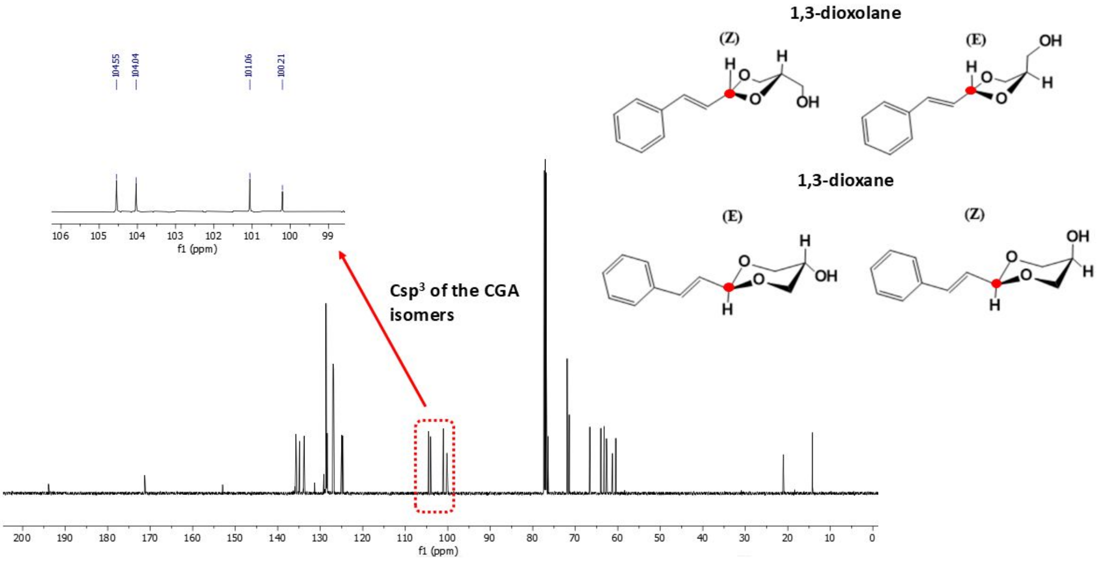

Figure S41: ^{13}C NMR of spectrum of CGA isomers mixture, isolated from the acetalization reaction of glycerol with cinnamaldehyde.

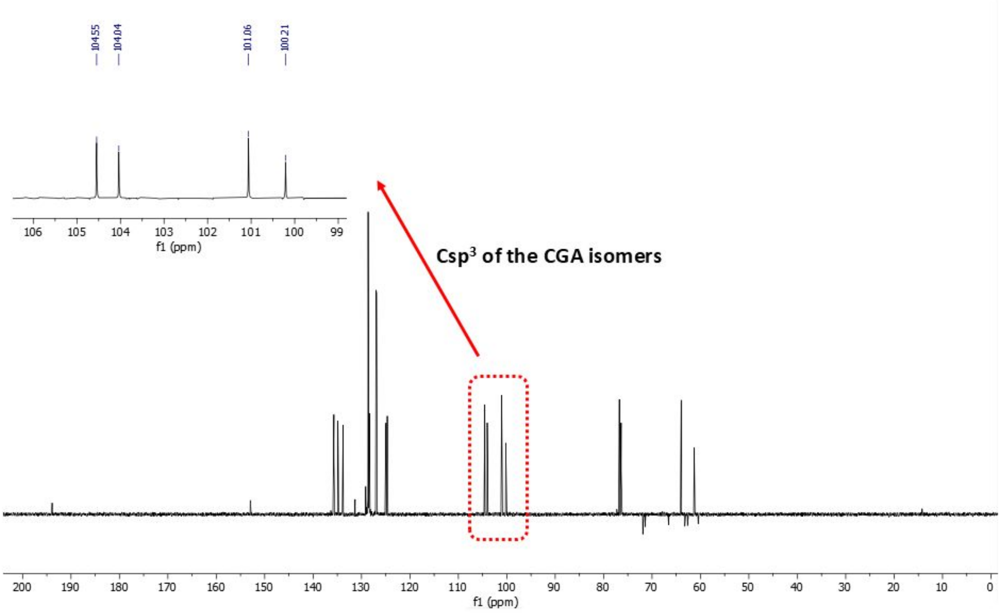

Figure S42: DEPT 90 ^{13}C NMR spectrum of CGA isomers mixture, isolated from the acetalization reaction of glycerol with cinnamaldehyde.

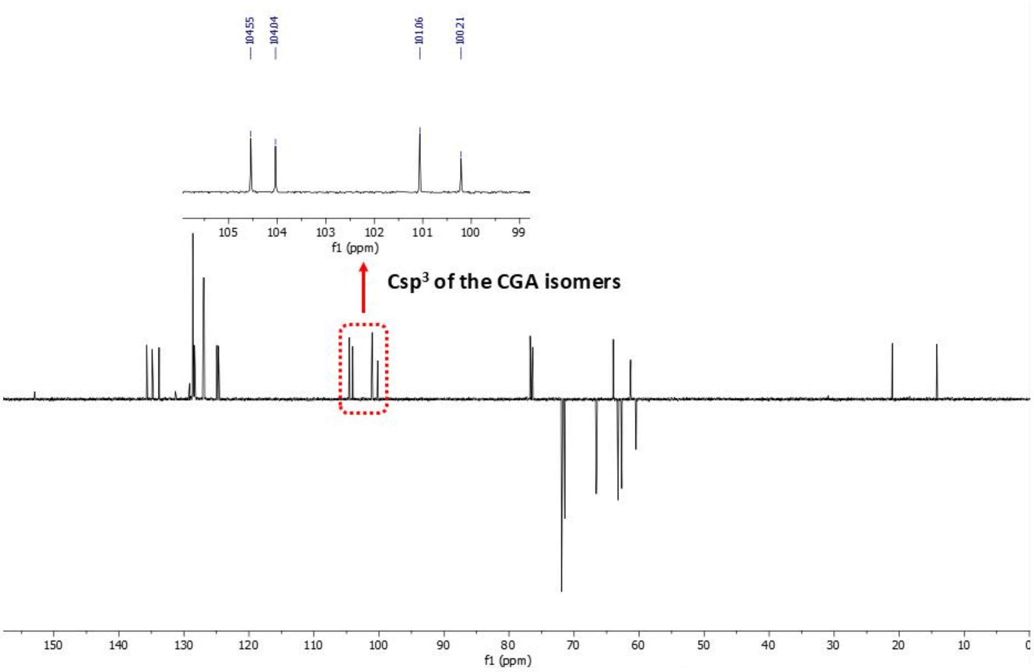

Figure S43: DEPT 135 ^{13}C NMR spectrum of CGA isomers mixture, isolated from the acetalization reaction of glycerol with cinnamaldehyde.
